# GeTallele: A Method for Analysis of DNA and RNA Allele Frequency Distributions

**DOI:** 10.3389/fbioe.2020.01021

**Published:** 2020-09-16

**Authors:** Piotr Słowiński, Muzi Li, Paula Restrepo, Nawaf Alomran, Liam F. Spurr, Christian Miller, Krasimira Tsaneva-Atanasova, Anelia Horvath

**Affiliations:** ^1^Department of Mathematics, College of Engineering, Mathematics and Physical Sciences, Living Systems Institute, Translational Research Exchange @ Exeter and The Engineering and Physical Sciences Research Council Centre for Predictive Modelling in Healthcare, University of Exeter, Exeter, United Kingdom; ^2^McCormick Genomics and Proteomics Center, School of Medicine and Health Sciences, The George Washington University, Washington, DC, United States; ^3^Department of Genetics and Genomics Sciences, Icahn School of Medicine at Mount Sinai, New York, NY, United States; ^4^Cancer Program, Broad Institute of MIT and Harvard, Cambridge, MA, United States; ^5^Medical Oncology, Dana-Farber Cancer Institute, Boston, MA, United States; ^6^Biological Sciences Division, Pritzker School of Medicine, The University of Chicago, Chicago, IL, United States; ^7^Department of Bioinformatics and Mathematical Modelling, Institute of Biophysics and Biomedical Engineering, Bulgarian Academy of Sciences, Sofia, Bulgaria; ^8^Department of Pharmacology and Physiology, School of Medicine and Health Sciences, The George Washington University, Washington, DC, United States; ^9^Department of Biochemistry and Molecular Medicine, School of Medicine and Health Sciences, The George Washington University, Washington, DC, United States

**Keywords:** variant allele fraction (VAF), RNA—DNA, earth mover's distance (EMD), circos plot, farey sequence

## Abstract

Variant allele frequencies (VAF) are an important measure of genetic variation that can be estimated at single-nucleotide variant (SNV) sites. RNA and DNA VAFs are used as indicators of a wide-range of biological traits, including tumor purity and ploidy changes, allele-specific expression and gene-dosage transcriptional response. Here we present a novel methodology to assess gene and chromosomal allele asymmetries and to aid in identifying genomic alterations in RNA and DNA datasets. Our approach is based on analysis of the VAF distributions in chromosomal segments (continuous multi-SNV genomic regions). In each segment we estimate variant probability, a parameter of a random process that can generate synthetic VAF samples that closely resemble the observed data. We show that variant probability is a biologically interpretable quantitative descriptor of the VAF distribution in chromosomal segments which is consistent with other approaches. To this end, we apply the proposed methodology on data from 72 samples obtained from patients with breast invasive carcinoma (BRCA) from The Cancer Genome Atlas (TCGA). We compare DNA and RNA VAF distributions from matched RNA and whole exome sequencing (WES) datasets and find that both genomic signals give very similar segmentation and estimated variant probability profiles. We also find a correlation between variant probability with copy number alterations (CNA). Finally, to demonstrate a practical application of variant probabilities, we use them to estimate tumor purity. Tumor purity estimates based on variant probabilities demonstrate good concordance with other approaches (Pearson's correlation between 0.44 and 0.76). Our evaluation suggests that variant probabilities can serve as a dependable descriptor of VAF distribution, further enabling the statistical comparison of matched DNA and RNA datasets. Finally, they provide conceptual and mechanistic insights into relations between structure of VAF distributions and genetic events. The methodology is implemented in a Matlab toolbox that provides a suite of functions for analysis, statistical assessment and visualization of Genome and Transcriptome allele frequencies distributions. GeTallele is available at: https://github.com/SlowinskiPiotr/GeTallele.

## Introduction

RNA and DNA carry and present genetic variation in related yet distinct manners; the differences encoding information about functional and structural traits. In diploid organisms, an important measure of genetic variation is the variant allele frequency (VAF), which can be measured from both genomic (DNA) and transcriptomic (RNA) sequencing data as the encoded and expressed allele frequencies, respectively. Differential DNA-RNA allele frequencies are associated with a variety of biological processes, such as genome admixture, and allele-specific transcriptional regulation (Ha et al., 2012; Shah et al., 2012; Han et al., 2015; Ferreira et al., 2016; Movassagh et al., 2016).

RNA-DNA allele comparisons from sequencing have mostly been approached at the nucleotide level, where they have proven to be highly informative for determining the allelic functional consequences (ENCODE Project Consortium, 2012; Ha et al., 2012; Shah et al., 2012; Morin et al., 2013; Han et al., 2015; Ferreira et al., 2016; Macaulay et al., 2016; Movassagh et al., 2016; Reuter et al., 2016; Shi et al., 2016; Shlien et al., 2016; Yang et al., 2016). Comparatively, integration of allele signals at the molecular level, as derived from linear DNA and RNA, is less comprehensively explored due to the challenges presented by limited compatibility of the outputs from the two sequencing assays.

Herein, we introduce a novel methodology for the analysis of DNA and RNA VAF distributions. This methodology is motivated by the following observations that, to our knowledge, have not been integrated into existing VAF analysis methodologies:

1. VAF distributions can change along a chromosome and differ between chromosomal segments (continuous multi-SNV genomic regions);
2. VAF distribution in a chromosomal segment is approximately symmetric;
3. VAF distribution in a chromosomal segment is a reflection of contributions from all the genetic events in all of the cells constituting the sequenced sample;
4. the variant and reference read counts can be modeled as random numbers from a binomial distribution; and
5. the support of the VAF distributions is a Farey sequence.

Guided by the first three observations, our methodology is designed to provide an aggregate description of VAF distribution in chromosomal segments.

The fourth observation motivates the development of a stochastic model for generating synthetic VAF samples. The model is a binomial mixture model, meaning that each of the mixture components is a binomial distribution parametrised by probability of success given a number of trials. The probability of success is equal across all binomial distributions in the mixture, while the number of trials varies between the mixture components. Each individual component has number of trials that is sampled from the total read counts in the dataset. We interpret the random numbers from this binomial mixture model as the number of variant reads at individual SNV loci. Namely, the common probability of success becomes variant probability, or v_PR_, defined as the probability of observing a variant allele at any site in a given chromosomal segment. We sample the total read counts from the data to account for technical variance arising from the sequencing process. The binomial mixture model implies that a variant or reference read at a given site is a result of a Bernoulli process.

Finally, the fifth observation allows for the rigorous comparison of observed and synthetic VAF distributions, resulting in the estimation of v_PR_ of observed VAF distributions.

The potential benefits of the proposed approach are 2-fold: first, by exploiting the statistical relations between SNVs in chromosomal segment, v_PR_ is less dependent on read depth and hence can help to utilize sequencing signals more efficiently; second, since v_PR_ is a high-level descriptor of VAF distributions, it allows for the direct comparison of DNA and RNA VAF distributions without the effects of limited comparability of DNA and RNA sequencing data.

## Materials and Methods

### Data

We evaluate and demonstrate GeTallele's functionality using matched whole exome and RNA sequencing datasets from paired normal and tumor tissue obtained from 72 female patients with breast invasive carcinoma (BRCA) from TCGA. Each dataset contains four matched sequencing sets: normal exome (Nex), normal transcriptome (Ntr), tumor exome (Tex), and tumor transcriptome (Ttr) (see Supplementary Table 1). The raw sequencing data were processed as previously described (Movassagh et al., 2016) to generate the inputs for GeTallele.

In short, all datasets were generated through paired-end sequencing on an Illumina HiSeq platform. The human genome reference (hg38)-aligned sequencing reads (Binary Alignment Maps, bams) were downloaded from the Genomic Data Commons Data Portal (https://portal.gdc.cancer.gov/) and processed downstream through an in-house pipeline. After variant calling (Li, 2011), the RNA-seq and whole exome sequencing (WES) alignments, together with their respective variant calls, were processed through the read count module of the package RNA2DNAlign (Movassagh et al., 2016), to produce variant and reference sequencing read counts for all the variant positions in all four sequencing signals (normal exome, normal transcriptome, tumor exome and tumor transcriptome). Selected read count assessments were visually examined using the Integrative Genomics Viewer (Thorvaldsdóttir et al., 2013).

For each sample, to select SNV positions for analysis, we start with heterozygous SNV calls in the normal exome (Li et al., 2009). In each of these positions, we estimate the counts of the variant and reference reads (n_VAR_ and n_REF_, respectively) across the 4 matching datasets, and retain positions covered by a minimum total (variant + reference) read depth for further analyses. This threshold is flexible and is required to ensure that only sufficiently covered positions will be analyzed; it is set to 3 in the herein presented results. For further analysis (without loss of generality), we transform all the original VAF values to VAF = |VAF−0.5|+0.5. We introduce this transformation due to the symmetric nature of the VAF distributions.

In addition, we required each tumor sample to have at least three of the following five purity estimates—Estimate, Absolute, LUMP, IHC, and the consensus purity estimate (CPE) (Katkovnik et al., 2002; Pagès et al., 2010; Carter et al., 2012; Yoshihara et al., 2013; Zheng et al., 2014; Aran et al., 2015). On the same datasets, we applied THetA (Oesper et al., 2013, 2014)—a popular tool for assessing CNA and admixture from sequencing data—was also applied to the datasets.

### Statistics

To test statistical significance, GeTallele uses parametric and non-parametric methods and statistical tests (Hollander et al., 2013; Corder and Foreman, 2014). Namely, to compare distributions of the variant allele frequencies (VAF) we use the Kolmogorov–Smirnov test (examples of VAF distributions are depicted in **Figures 2**, **3**). To study concurrence of windows, we use permutation/bootstrap tests. To test relations between v_PR_ and copy number alterations (CNA), we use Pearson's correlation coefficient.

To account for multiple comparisons, we set the probability for rejecting the null hypothesis at *p* < 1e−5, which corresponds to Bonferroni (Dunn, 1961) family-wise error rate (FWER) correction against 100,000 comparisons. We use a fixed value, rather than other approaches, to ensure better consistency and reproducibility of the results. Alternatively, we apply Benjamini and Hochberg (Benjamini and Hochberg, 1995) false discovery rate (FDR) correction with a probability of accepting false positive results p_FDR_ <0.05. We specify the method used in the text when reporting the results.

## Description of the Novel Methodology

**Figure 1:**
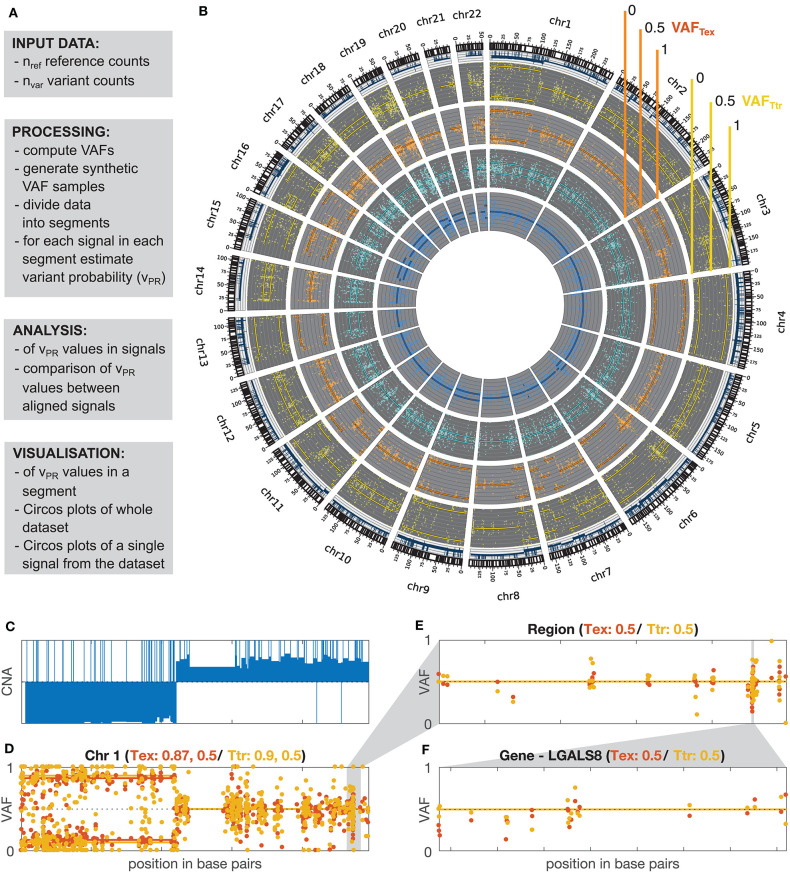
GeTallele and visualization of VAF data. **(A)** Toolbox description. **(B)** Visualization of the whole dataset on the level of genome using Circos plot (blue, normal exome; cyan, normal transcriptome; orange, tumor exome; yellow, tumor transcriptome). **(C)** CNA values for chromosome 1. **(D–F)** Visualization of the VAF values with fitted variant probability (v_PR_–see section Estimation of Variant Probability v_PR_ and Figure 3). VAF_TEX_ and VAF_TTR_ values at the level of: chromosome (chromosome 1) **(D)**, custom genome region **(E)**, and gene **(F)**. **(D)** Shows that there are two chromosomal segments with different VAF distributions, likely representing a region of copy-neutral loss of heterozygosity. **(C)** Shows that large scale change in the CNA is concurrent with the change in the VAF distributions. In panel titles: Tex, v_PR_ estimate for VAF distributions of tumor exome (orange); Ttr, v_PR_ estimate for VAF distributions of tumor transcriptome (yellow).

**Figure 2:**
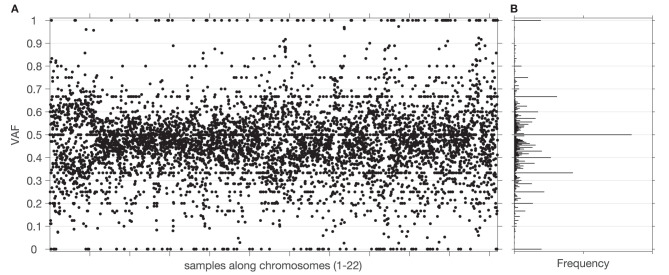
Sample and distribution of variant allele frequencies (VAF) values. **(A)** All the VAF values of a tumor exome sequencing signal (chromosomes 1–22) from one of the datasets. **(B)** Histogram of the VAF values from **(A)**. Centers of bins of the histogram are located at elements of a Farey sequence.

The overall workflow of the proposed methodology as implemented in the GeTallele is shown in Figure 1. As input, GeTallele requires the absolute number of sequencing reads bearing the variant and reference nucleotide in each single-nucleotide variant (SNV) position. For each available dataset (4 in the presented analysis) GeTallele estimates VAF based on the variant and reference reads (n_VAR_ and n_REF_, respectively) covering the positions of interest: VAF = n_VAR_/(n_VAR_ + n_REF_). An example of genome-wide VAF values estimated from tumor exome Tex dataset, and their corresponding histogram is shown in Figure 2.

### Data Segmentation

To analyse variant allele frequencies (VAF) at genome-wide level, GeTallele first divides the VAF sequence into a set of non-overlapping segments along the chromosomes. To partition the data into segments, GeTallele uses a parametric global method, which detects the breakpoints in a signal using its mean, as implemented in the Matlab function findchangepts (Lavielle, 2005; Killick et al., 2012) In each segment, the VAFs of the chosen signal must have a different mean than that of the adjacent segment. In the Matlab implementation, sensitivity of breakpoint detection can be controlled using parameter MinThreshold; with a default setting of 0.2. Segments containing fewer than 10 data points were merged with the preceding segment. For analysis of matched signals, segmentation is based on one signal, and then applied to the others. In the presented analysis, segmentation is based mainly on Tex dataset, for comparison we also use Ttr dataset (dataset used for segmentation is specified in the description of the results presented in Section Results).

### Estimation of Variant Probability v_PR_

Variant probability is a biologically interpretable quantitative descriptor of the VAF distribution. It is the common probability of observing a variant allele at any site in a given chromosomal segment. The v_PR_ is a measure describing the genomic event that, through the sequencing process, was transformed into an observed distribution of VAFs. For example, in VAF_DNA_ from a diploid genome, we assume variant probability v_PR_ = 0.5 (meaning that both alleles are equally probable) corresponds to a true allelic ratio of 1:1 for heterozygous sites. The value might differ from 0.5 due to reference mapping biases (Degner et al., 2009). For heterozygous sites in the DNA from a diploid monoclonal samples, the corresponding tumor VAF_DNA_ is expected to have the following interpretations: v_PR_ = 1 or v_PR_ = 0 corresponding to a monoallelic status resulting from a deletion, and v_PR_ = 0.8 (or 0.2), 0.75 (or 0.25), 0.67 (or 0.33) corresponding to allele-specific tetra-, tri-, and duplication of the variant-bearing allele, respectively.

The v_PR_ of the VAF_RNA_ is interpreted as follows. In positions corresponding to heterozygote sites in DNA, alleles not preferentially targeted by regulatory traits are expected to have expression rates with variant probability v_PR_ = 0.5, which (by default) scale with the DNA allele distribution. Differences between VAF_DNA_ and VAF_RNA_ values are observed in special cases of transcriptional regulation where one of the alleles is preferentially transcribed over the other. In the absence of allele-preferential transcription, VAF_DNA_, and VAF_RNA_ are anticipated to have similar v_PR_ across both diploid (normal) and copy number altered genomic regions. Consequently, VAF_DNA_, and VAF_RNA_ are expected to synchronously switch between allelic patterns along the chromosomes, with the switches indicating breakpoints of DNA deletions or amplifications.

Since we observed that DNA and RNA signals have different distributions of total reads and also that the distributions of total reads vary between participants, the synthetic VAF distributions are generated individually for each sequencing signal and each participant.

To estimate v_PR_ in the signals, GeTallele first generates synthetic VAF distributions and then uses the earth mover's distance (EMD) (Kantorovich and Rubinstein, 1958; Levina and Bickel, 2001) to fit them to the data. To generate a synthetic VAF distribution with a given variant probability, v_PR_, GeTallele, bootstraps 10,000 values of the total reads (sum of the variant and reference reads; n_VAR_ + n_REF_) from the analyzed signal in the dataset. It then uses binomial pseudorandom number generator to get number of successes for given number of total reads and a given value of v_PR_ (implemented in the Matlab function binornd). The v_PR_ is the common value of the probability of success and generated number of successes is interpreted as an n_VAR_. Since the v_PR_ of the synthetic sample can take any value, it can correspond to a single genomic event as well as any combination of genomic events in any mixture of normal and tumor populations (See section v_PR_ Values in Mixtures of Normal and Tumour Populations).

**Figure 3:**
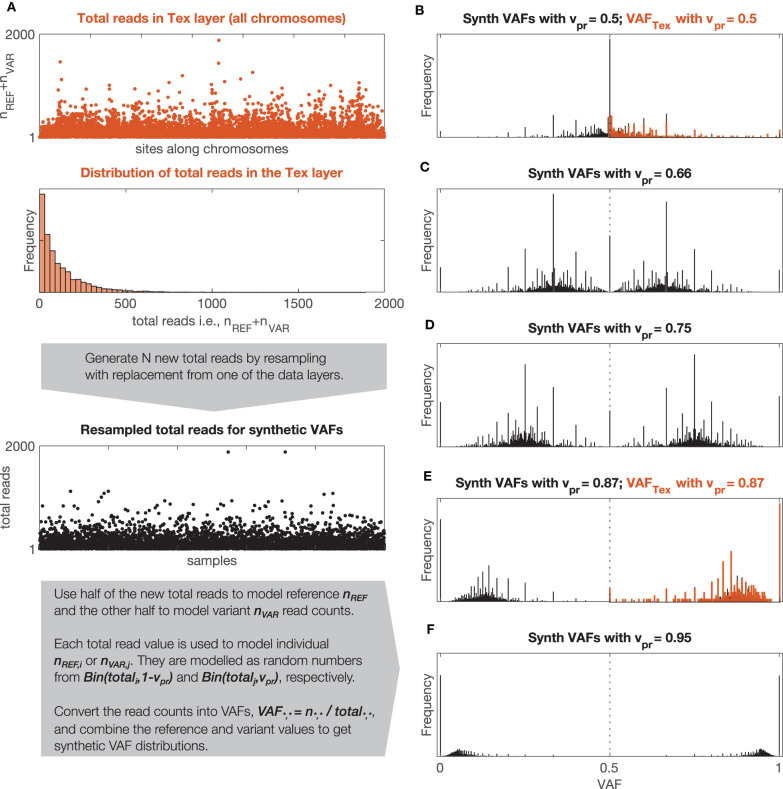
Synthetic and observed VAF distributions. **(A)** Description of the model used to generate synthetic VAF distributions, Bin(n,p) stands for binomial distribution with parameters n (number of trials) and p (probability of success). **(B–F)** Synthetic VAF distributions for different values of v_PR_. **(B,F)** Show additionally distributions of VAF_TEX_ for the two windows shown in Figure 1D.

The analysis presented in the paper uses 51 synthetic VAF distributions with v_PR_ values that vary from 0.5 to 1 with step (increment of) 0.01. The synthetic VAF distributions are parametrized using only v_PR_ ≥ 0.5, however, to generate them we use v_PR_ and its symmetric counterpart 1-v_PR_. The process of generating synthetic VAF distributions along with examples of synthetic and real VAF distributions with different values of v_PR_ are illustrated in Figure 3.

To estimate v_PR_, we compute the Earth mover's distance between the distribution of VAF values in the considered window and the 51 synthetic VAF distributions (i.e., observed vs. synthetic VAF). The estimate is given by the v_PR_ of the synthetic VAF distribution that is closest to the VAF distribution in the segment.

Earth mover's distance (EMD) is a metric for quantifying differences between probability distributions (Kantorovich and Rubinstein, 1958; Levina and Bickel, 2001) and in the case of univariate distributions it can be computed as:

$$\begin{array}{l} \text{EMD}\left(\text{PD}{\text{F}}_{1},\text{PD}{\text{F}}_{2}\right)=\int_{Z}|CDF_{1}\left(z\right)CDF_{2}\left(z\right)|dz. \end{array}$$

Here, PDF_1_ and PDF_2_ are two probability density functions, and CDF_1_ and CDF_2_ are their respective cumulative distribution functions. Z is the support of the PDFs (i.e., set of all the possible values of the random variables described by them). Because VAFs are defined as simple fractions with values between 0 and 1, their support is given by a Farey sequence (Hardy and Wright, 2008) of order n; n is the highest denominator in the sequence. For example, Farey sequence of order 2 is 0, 1/2, 1, and Farey sequence of order 3 is 0, 1/3, 1/2, 2/3, 1. We use a Farey sequence of order 1,000 as the support Z for estimating the v_PR_.

**Figure 4:**
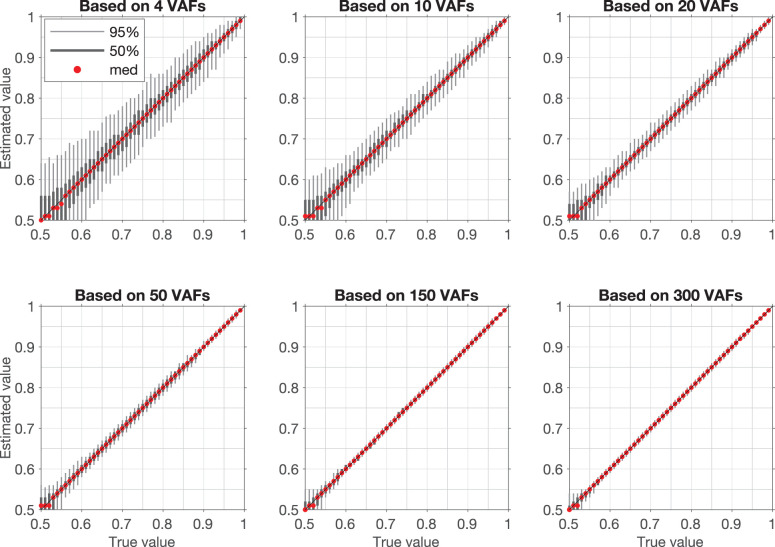
Confidence intervals for artificial samples with different numbers of VAFs. Each confidence interval is based on estimation of v_PR_ in 1,000 randomly generated samples with a fixed v_PR_ (True value). Light gray bar is 95% confidence interval (950 samples lay within this interval), dark gray bar is 50% confidence interval (500 samples lay within this interval), red dot is median value.

Examples of VAF distributions with fitted synthetic VAF distributions are shown in Figures 3A,D. The dependence of the confidence intervals of the estimation on the number of VAF values in a segment is illustrated in Figure 4, which clearly demonstrates that the accuracy of the estimate is positively correlated with the number of VAFs in the chosen segment.

### v_PR_ Values in Mixtures of Normal and Tumor Populations

Since the v_PR_ can take any value between 0.5 and 1 it can correspond to a single genomic event as well as any combination of genomic events in any mixture of normal and tumor populations. A mixture v_PR_ value that corresponds to a combination of genomic events can be computed using the following expression:

$${\text{v}}_{\text{PR}}=\frac{\sum_{\text{pl}=1}^{\text{pl}=\text{N}}\sum_{{\text{e}}_{\text{VAR}}=\{\text{events}\}}{\text{e}}_{\text{VAR}}\cdot {\text{p}}_{\text{PL}}}{\sum_{\text{pl}=1}^{\text{pl}=\text{N}}\sum_{{\text{e}}_{\text{VAR}}=\{\text{events}\}}{\text{e}}_{\text{VAR}}\cdot {\text{p}}_{\text{PL}}+\sum_{\text{pl}=1}^{\text{pl=N}}\sum_{{\text{e}}_{\text{REF}}=\{\text{events}\}}{\text{e}}_{\text{REF}}\cdot {\text{p}}_{\text{PL}}}$$

Where e_VAR_ and e_REF_ are the multiplicities of variant and reference alleles and p_PL_ is a proportion of one of the populations. For heterozygote sites e_VAR_ = 1 and e_REF_ = 1, for deletions e_VAR_ = 0 or e_REF_ = 0, for du-, tri- and tetraplications e_VAR_ or e_REF_ can be equal to 2, 3 or 4, respectively. The sum of proportions p_PL_ over the populations is equal 1. For example, for a mixture of 1 normal (N, p_N_ = 0.44) and 2 tumor populations (T1, p_T1_ = 0.39 and T2, p_T2_ = 0.17), T1 with deletion and T2 with deletion the mixture v_PR_ value can be computed as follows:

$$\begin{array}{l} {\text{ v}}_{\text{PR}}=\frac{{\text{p}}_{\text{N}}\cdot \text{B}+{\text{p}}_{\text{T}1}\cdot \text{B}+{\text{p}}_{\text{T}2}\cdot \text{B}}{{\text{p}}_{\text{N}}\cdot \left(\text{A}+\text{B}\right)+{\text{p}}_{\text{T}1}\cdot \left(0+\text{B}\right)+{\text{p}}_{\text{T}2}\cdot \left(0+\text{B}\right)}\\ =\frac{0.44\cdot 1+0.39\cdot 1+0.17\cdot 1}{0.44\cdot \left(1+1\right)+0.39\cdot \left(1+0\right)+0.17\cdot \left(1+0\right)}=0.694. \end{array}$$

**Figure 5:**
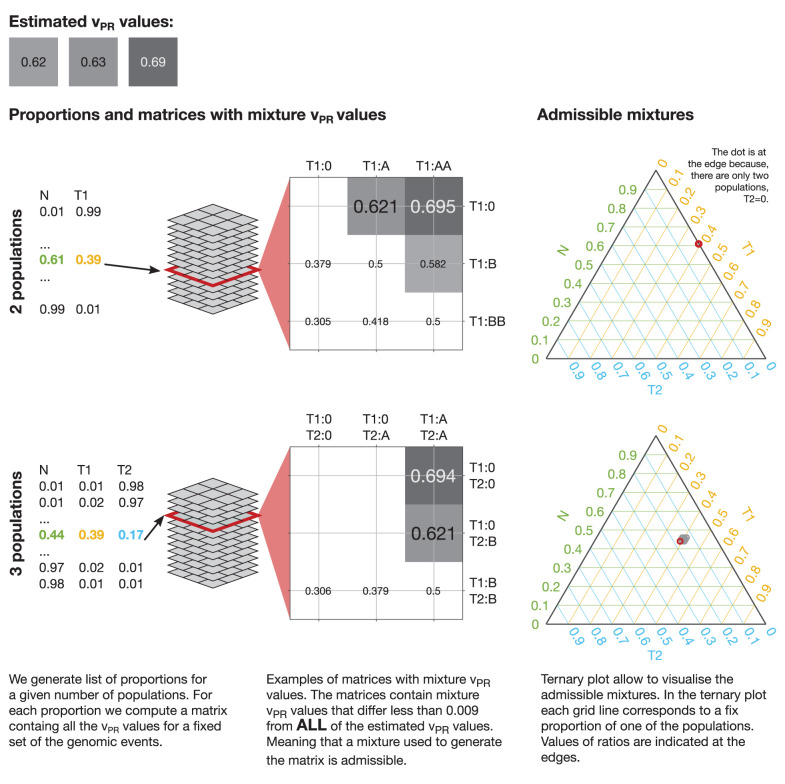
Mixtures admissible by the v_PR_ values estimated from data. To uncover mixtures that could produce the three estimated v_PR_ values we perform an exhaustive approximate search of all the possible v_PR_ values produced by any mixture of the populations with a given set of genetic events. In each case we generate a full set of proportions with a given step (e.g., 0.01) and compute all the possible v_PR_ values that such a mixture could produce. In the illustrated cases: 2 populations (1 tumor) could produce the estimated v_PR_ values through a deletion (estimated v_PR_ = 0.62 and v_PR_ = 0.63) and via deletion of one allele and duplication of another (estimated v_PR_ = 0.69); 3 populations (2 tumors) could produce the estimated v_PR_ values through a deletion in one of the tumor populations (estimated v_PR_ = 0.62 and v_PR_ = 0.63) and via deletion in both of the tumor populations (estimated v_PR_ = 0.69). The 2 populations case admits a single mixture and the 3 populations allow 9 mixtures with similar compositions. The admissible mixtures are depicted on the ternary plots, red circle indicates solution corresponding to the presented matrix. We exclude mixture v_PR_ values that result from deletion of both the variant and reference alleles (empty fields in the matrices).

By comparing the v_PR_ values estimated from data with possible mixture v_PR_ values we propose to estimate sample purity and its clonal composition. To this end, we first generate a full set of proportions of all the population in the mixture with step (increment of) 0.01 and compute all the possible v_PR_ values that each of the mixtures could produce. For step 0.01: two populations (1 tumor) give 99 proportions, three populations (2 tumors) give 4,851 proportions, four populations (3 tumors) give 156,849 proportions. The matrices with mixture v_PR_ values for each proportion, vary from 2 × 2, for two populations with deletions, to 35 × 35 for four populations with all events up to tetra-plications. Then, we run an exhaustive approximate search over all the matrices with mixture v_PR_ values over all the proportions. The search is approximate because the estimated v_PR_ values have limited accuracy and because we consider only discrete values of proportions. In the analysis we define a match between estimated and mixture v_PR_ values if they differ by <0.009 (we chose a value that is smaller than the smallest difference between possible v_PR_ estimates). The search returns a large number of admissible mixtures that could produce the estimated v_PR_ values. This process is illustrated in Figure 5.

**Figure 6:**
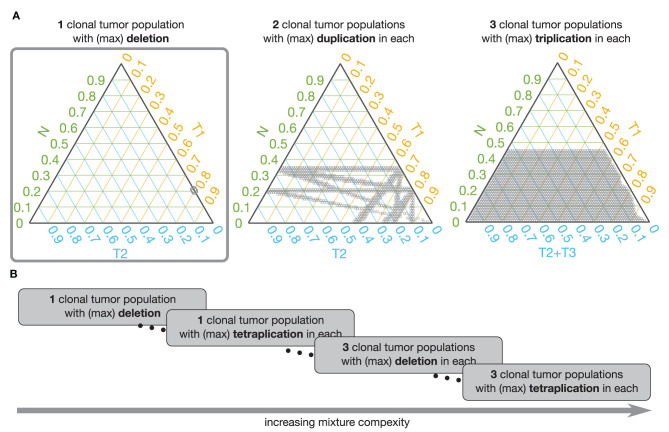
Admissible mixtures for increasing mixture complexity. **(A)** Shows admissible mixtures for 3 different mixtures with increasing complexity. The simplest mixture (mixture with the lowest number of components and the simplest set of genetic events) is show within the gray frame. On each ternary plot, the admissible mixtures are indicated by gray dots. The green axis indicates proportion of the normal population (N), the yellow axis indicates proportion of the 1st tumor population (T1), the blue axis shows proportion of the 2nd tumor population (T2), or sum of the 2nd and 3rd tumor populations (T2 + T3). **(B)** Schematic representation of increasing complexity of the mixture models. From a mixture of 1 normal and 1 tumor population in which only deletion is possible to a model with 1 normal and 3 tumor populations and each can have deletions, du-, tri-, and tetraplications.

To visualize the admissible mixtures, we use ternary plots, which allow us to illustrate composition of three components in two dimensions. The composition, represented by ratios of the three components, which sum to a constant, is depicted as point inside or on the edge of an equilateral triangle. If the point is on the edges, the composition has only two components. To help interpretation of the ternary plots, we also plot the grid lines that are parallel to the sides of the triangle. These gridlines indicate the directions of constant ratios of the components. Along such direction the ratio of one of the components is fixed and only the other two ratios vary. Examples of visualization of admissible mixtures on ternary plots are shown in Figures 5, 6.

To facilitate analysis of the admissible mixtures returned by the search procedure we introduce mixture complexity. Mixture complexity is a measure that increases with number of populations as well as with variety of genetic events. From the simplest mixture of 1 normal and 1 tumor population in which only deletions are possible to a model with 1 normal and multiple tumor populations where each can have deletions, and any level of multiplications. In practice, we set the limit at 3 tumor populations and tetra-plications. Mixture complexity helps to group and visualize admissible mixtures. Mixtures with higher complexity allow more possible v_PR_ values, meaning that it is easier to find the match with the estimated v_PR_ values but that the number of admissible mixtures increases (see Figure 6). We, further, observe that proportion of normal population, p_N_, increases with a number of clonal tumor populations included in the model mixture and that, generally, p_N_ stays constant with increasing variety of genetic events, for a fixed number of clonal tumor populations. We note that this is just one of many possible ways of deciding which solution should be chosen.

## Results

To evaluate the proposed methodology, we apply it on matched normal and tumor exome and transcriptome sequencing data of 72 breast carcinoma (BRCA) datasets with pre-assessed copy-number and genome admixture estimates acquired through TCGA (see Materials and Methods). We first compare DNA and RNA VAF distributions from matched sequencing datasets and find that both genomic signals give very similar results in terms of segmentation and estimated variant probability values. We further assess the correlations between v_PR_ values and copy number alterations (CNA) values and find that they are in agreement with each other. Finally, we use the v_PR_ values to estimate tumor purity. The purity estimates based on v_PR_ values show good concordance with alternative approaches.

### Segmentation Results

Segmentation of the data, based on the tumor exome signal, resulted in 2,697 chromosomal segments across the 72 datasets. We excluded from further analysis 294 chromosomal segments where either tumor exome or transcriptome had v_PR_ > = 0.58 but their VAF distribution could not be differentiated from the model VAF distributions with v_PR_ = 0.5 (*p* > 1e−5, Kolmogorov Smirnov test, equivalent to Bonferroni FWER correction for 100,000 comparisons). The 294 excluded chromosomal segments, corresponding to 4% of the total length of the data in base pairs and 4% of all the available data points. This implies these short segments containing few VAF values. In the remaining 2,403 chromosomal segments, we systematically examined the similarity between corresponding VAF_TEX_ (tumor exome), VAF_TTR_ (tumor transcriptome), and CNA. We obtained several distinct patterns of coordinated RNA-DNA allelic behavior as well as correlations with CNA data.

In 60% of all analyzed chromosomal segments the distributions of VAF_TEX_ and VAF_TTR_ were statistically concordant (*P* > 1e−5, Kolmogorov Smirnov test), and in 40% they were statistically discordant (*P* < 1e−5, Kolmogorov Smirnov test). In two chromosomal segments, VAF_TEX_ and VAF_TTR_, had the same v_PR_, while having statistically different VAF distributions (P < 1e−5, Kolmogorov Smirnov test). We consider such chromosomal segments as concordant. The v_PR_ robustly characterizes VAF sample while the Kolmogorov-Smirnov test is very sensitive for differences between distributions that might be caused by to technical variance. In the vast majority of the discordant chromosomal segments v_PR_ of the VAF_TTR_, v_PR,TTR_, was higher than v_PR_ of the VAF_TEX_, v_PR,TEX_, (only in 21 out of 959 discordant chromosomal segments v_PR,TTR_ was lower than v_PR,TEX_).

### Concurrence of Segmentation Based on DNA and RNA

We next analyzed the concurrence between chromosomal segments resulting from independent segmentations of the tumor exome (VAF_TEX_) and transcriptome (VAF_TTR_) datasets (2,697 and 3,605 chromosomal segments, respectively, across all the samples). We first assessed chromosome-wise alignment of the start and end points of the chromosomal segments. In 45% of the chromosomes both VAF_TEX_ and VAF_TTR_ signals produce a single segment that contains the whole chromosome. In 33% of chromosomes both signals produced multiple chromosomal segments. These chromosomal segments are well aligned, with 90% of the breakpoints differing <7% of data points in the chromosome, e.g., they are <70 points apart if the chromosome contains 1,000 data points; Q50 = 0.02%, Q75 = 2% of data points in the chromosome. The probability of observing such an alignment by chance is smaller than *p* = 1e−5 (100,000 bootstrap samples with breakpoints assigned randomly in all the individual chromosomes where both signals produced multiple chromosomal segments). In 22% of the chromosomes, segments based on VAF_TEX_ and VAF_TTR_ signals were positionally discordant—one signal produced a single segment containing whole chromosome while the other produced multiple chromosomal segments.

**Figure 7:**
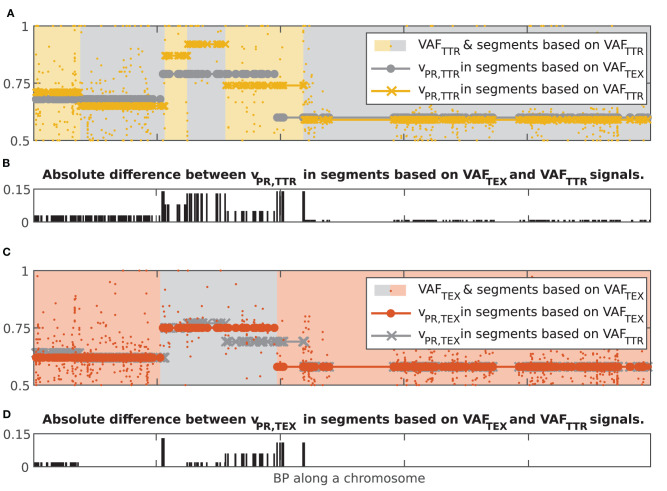
Illustration of concurrence between chromosomal segment resulting from independent segmentations of the dataset based on the VAF_TEX_ and VAF_TTR_ signals. **(A)** Yellow dots, VAF_TTR_; gray circles, v_PR,TTR_ interpolated at all data points in segments based on VAF_TEX_; yellow crosses, v_PR,TTR_ interpolated at all data points in segments based on VAF_TTR_. **(B)** Bar plot of the absolute difference between the v_PR_ values in the two kinds of chromosomal segments. **(C)** orange dots, VAF_TEX_; gray crosses, v_PR,TEX_ interpolated at all data points in segments based on VAF_TEX_; orange dots v_PR,TEX_ interpolated at all data points in segments based on VAF_TTR_. **(D)** Bar plot of the absolute difference between the v_PR_ values in the two kinds of chromosomal segments.

To compare the v_PR_ values in the 55% of chromosomes where at least one signal produced more than one chromosomal segment, we computed chromosome-wise mean absolute error (MAE) between the v_PR_ in two sets of chromosomal segments. To account for different start and end points of the segments we interpolated the v_PR_ values (nearest neighbor interpolation) at each data point in the chromosome. We separately compared the v_PR,TEX_ and v_PR,TTR_ values. Assessment of alignment using MAE showed strong concordance: v_PR,TEX_ agreed perfectly in 11% of the chromosomes and had the percentiles of MAE equal to Q50 = 0.012, Q75 = 0.022 and Q97.5 = 0.047, while v_PR,TTR_ agreed perfectly in 8% but had slightly higher percentiles of MAE Q50 = 0.019, Q75 = 0.034 and Q97.5 = 0.07. v_PR,TEX_ and v_PR,TTR_ values had MAE = 0 simultaneously in 4% of the chromosomes. Probability of observing such values of MAE by chance is smaller than *p* = 1e−3 (1,000 random assignments of v_PR,TEX_ and v_PR,TTR_ values to windows in the 873 chromosomes where at least one signal had more than one chromosomal segment). It is noteworthy that MAE Q97.5 < 0.07 is comparable with the confidence interval of single v_PR_ estimate based on 50 VAF values. In other words, both signals in a sample (Tex and Ttr) give very similar results in terms of segmentation and estimated values of the v_PR_. Albeit, segmentation of VAF_TTR_ generates a higher number of chromosomal segments. The higher number of VAF_TTR_ chromosomal segments indicates that transcriptional regulation occurs at a smaller scale than alterations in DNA. Figure 7 shows examples of concurrence between chromosomal segments based on VAF_TEX_ and VAF_TTR_ signals in a positionally concordant chromosome (both signals produced multiple segments).

### Correlation Between v_PR_ and CNA

Finally, we assess the correlations between v_PR_ and CNA in the individual samples. We separately computed correlations for deletions and amplifications. In order to separate deletions and amplifications, for each data set we found CNA_MIN_, value of the CNA in the range −0.3 to 0.3 that had the smallest corresponding v_PR,TEX_. To account for observed variability of the CNA values near the CNA_MIN_, we set the threshold for amplifications to CNA_AMPLIFICATION_ = CNA_MIN_-0.05, and for deletions we set it to CNA_DELETION_ = CNA_MIN_ + 0.05 (each data set had a different threshold).

For VAF_TEX_, we observed significant correlations with negative trend between v_PR,TEX_ and CNA ≤ CNA_DELETION_ in 57 datasets and with a positive trend between v_PR,TEX_ and CNA ≥ CNA_AMPLIFICATION_ in 39 datasets (p_FDR_ < 0.05, Pearson's correlation with Benjamini Hochberg multiple comparison correction for 72 samples). For VAF_TTR_, we observed significant correlations with a negative trend between v_PR,TTR_ and CNA ≤ CNA_DELETION_ in 62 datasets and with positive trend between v_PR,TTR_ and CNA ≥ CNA_AMPLIFICATION_ in 33 datasets (p_FDR_ < 0.05, Pearson correlation with Benjamini Hochberg correction). These correlations indicate that the segmentation and the estimated v_PR_ values are concordant with CNA calls. However, the v_PR_ values (estimated at the level of chromosomal segments) do not differentiate between positive and negative values of the CNA, meaning it is not possible to use v_PR_ alone to call amplifications and deletions.

**Figure 8:**
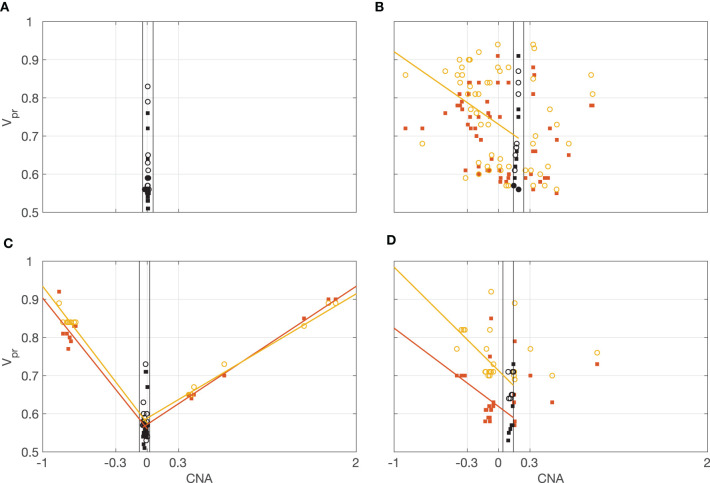
Illustration of the correlations between v_PR_ and CNA. Orange squares v_PR,TEX_, yellow circles v_PR,TTR_. Lines, least-squares fitted trends for significant correlations (orange correlation with v_PR,TEX_, yellow correlation with v_PR,TTR_). Black, v_PR_ for CNA_MIN_ ± 0.05. Correlations for all the datasets are shown in Supplementary Figure 1. **(A)** All the values of CNA are close to CNA_MIN_ = 0. **(B)** Relationship between CNA and v_PR_ is noisy, only some correlations are statistically significant. **(C)** All the correlations are statistically significant, v_PR,TTR_ values (circles) follow closely the v_PR,TEX_ (squares) indicating concordance of the VAF_TEX_ and VAF_TTR_ distributions. **(D)** Only correlations for CNA ≤ CNA_D_ are statistically significant.

Figure 8 shows four typical patterns of correlation between the CNA and v_PR_ values observed in the data. In Figure 8A, all the values of CNA are close to CNA_MIN_. In Figure 8B, the relationship between CNA and v_PR_ is noisy, only correlations between v_PR,TTR_ and CNA ≤ CNA_DELETION_ are statistically significant (r_TEX,CNA,DEL_ = −0.29, p_FDR_ = 0.063; r_TEX,CNA,DEL_ = −0.38, p_FDR_ = 0.012; r_TEX,CNA,AMPL_ = 0.14, p_FDR_ = 0.58; r_TEX,CNA,AMPL_ = 0.19, p_FDR_ = 0.47; Pearson's correlation with Benjamini Hochberg multiple comparison correction for 72 samples). In Figure 8C all the correlations are statistically significant, v_PR,TTR_ values (circles) follow closely the v_PR,TEX_ (squares) indicating that in most of the windows distributions of the VAF_TEX_ and VAF_TTR_ are concordant (r_TEX,CNA,D_ = −0.91, p_FDR_ < 1e−10; r_TEX,CNA,DEL_ = −0.96, p_FDR_ < 1e−10; r_TEX,CNA,AMPL_ = 0.92, p_FDR_ < 1e−10; r_TEX,CNA,AMPL_ = 0.95, p_FDR_ < 1e−10). In Figure 8D correlations between v_PR,TEX_, v_PR,TTR_ and CNA ≤ CNA_D_ are statistically significant, but there is a large difference (with median of 0.18) between v_PR,TEX_ and v_PR,TTR_ values, indicating that in most of the windows the distributions of the VAF_TEX_ and VAF_TTR_ in this dataset are discordant (r_TEX,CNA,DEL_ = −0.44, p_FDR_ = 0.047; r_TEX,CNA,DEL_ = −0.64, p_FDR_ = 0.0017; r_TEX,CNA,AMPL_ = 0.44 p_FDR_ = 0.16; r_TEX,CNA,AMPL_ = 0.28, p_FDR_ = 0.41). In many of the datasets we observe that the v_PR,TTR_ values are higher than the corresponding v_PR,TEX_ values (median v_PR,TTR_-v_PR,TEX_ = 0.03), likely indicative of preferential transcription of some alleles in the chromosomal segment. Correlations between v_PR_ and CNA in all datasets are shown in the Supplementary Figure 1.

### v_PR_ Based Purity Estimation

To demonstrate a practical application of the v_PR_ values we use them to estimate tumor purity of the samples. To this end we compared the v_PR_ based purity (VBP) estimates with ESTIMATE, ABSOLUTE, LUMP, IHC, and the Consensus Purity Estimation (CPE) (Katkovnik et al., 2002; Pagès et al., 2010; Carter et al., 2012; Yoshihara et al., 2013; Zheng et al., 2014; Aran et al., 2015).

To obtain the VBP estimate we used v_PR,TEX_ values. We, first, selected the v_PR,TEX_ values that: 1. are estimated with high confidence, i.e., are based on at least 50 VAF values; 2. are most likely heterozygous in normal exome, i.e., have a corresponding v_PR_ value in normal exome v_PR,NEX_ <0.58; 3. most likely have v_PR,TEX_ > 0.5, i.e., their *p-*value for comparison with v_PR,TEX_ = 0.5 is very small *p* < 1e−5 (Kolmogorov-Smirnov test).

Next, we used the selected v_PR,TEX_ values to find all admissible mixtures (with 1–3 tumor populations and allowing for all events, from deletions to tetraplications). To estimate the VBP, out of all the admissible mixtures we chose these with lowest mixture complexity and among these mixtures we take one with the highest p_N_ (proportion of the normal population). The VBP, percentage of tumor populations in the sample, is then given as 1-p_N_. Such approach provides rather conservative estimates of VBP (the smallest 1-p_N_). However, GetAllele can be extended to offer alternative methods of employing the admissible mixtures to estimate VBP. Development, analysis and comparison of alternative VBP estimation methods is beyond scope of the current paper.

**Figure 9:**
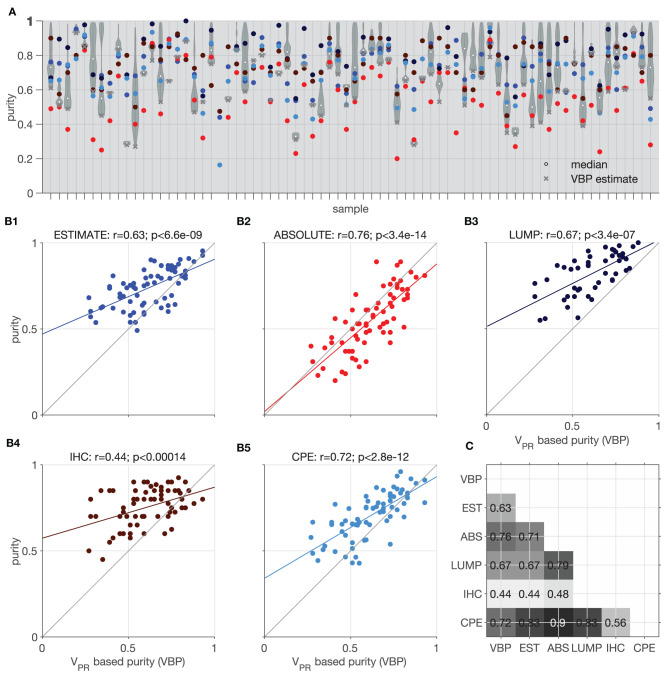
Illustration of purity estimation based on model mixtures and v_PR,TEX_. Comparison of the Estimate (EST), Absolute (ABS), LUMP, IHC, and the consensus purity estimate (CPE) methods with v_PR_ based purity (VBP). **(A)** Violin plots show distributions of purity based on all the admissible proportions of the normal population (x) indicates the lowest value selected as the most conservative estimate; colors corresponding to the different methods are indicated in **(B1–5)**. **(B1–5)** Correlation of the VBP with individual methods; colored line indicates best fit linear trend. **(C)** Matrix showing significant *p* < 0.05 Pearson's correlation coefficients between all tested methods.

Figure 9A shows violin plots of all considered 1-p_N_ values and (x) indicates the smallest value taken as a VBP estimate. In two of the datasets we could not estimate the purity due to lack of suitable v_PR,TEX_ values. The VBP estimates shows the best agreement with ABSOLUTE method (y = 0.86 x + 0.02, r = 0.76, *p* < 3.4e−14, Pearson's correlation, Figure 9B2). We suppose that this is because the ABSOLUTE method is based on copy number distributions, and our analysis (Section Correlation Between v_PR_ and CNA) revealed high correlations between the CNAs and v_PR_ values. Similar, to the ABSOLUTE method, VBP estimates are generally lower than the other purity estimates (ESTIMATE, LUMP, IHC, CPE); see Figures 9B1–5.

The approach presented in this section differs from other methods for inferring genomic mixture composition in that it is based on chromosomal segments with at least 50 VAF values which can extend over millions of base pairs. In contrast, PyClone (Roth et al., 2014) is based on sets of carefully selected individual deeply sequenced VAF values, while SciClone (Miller et al., 2014) and TPES (Locallo et al., 2019) are based on analysis of selected VAF values aggregated from the genome-wide sequences (multiple chromosomes). By using chromosomal segments, v_PR_ allows for a more granular description of the VAF distributions than aggregating genome-wide VAF values. At the same time, basing purity estimation on v_PR_ values allows for the use of SNVs with a low sequencing depth (3 in the presented analysis). Rigorous comparison of the performance of the different methods is beyond the scope of this demonstration of potential practical applications of v_PR_.

## Discussion

We present a novel methodology to assess allele asymmetries in RNA and DNA datasets using VAF. Simultaneous analysis of RNA and DNA VAF is becoming more feasible with the growing accessibility of paired RNA and DNA sequencing datasets from the same individual (ENCODE Project Consortium, 2012; Macaulay et al., 2016; Reuter et al., 2016). Our approach addresses the compatibility between RNA and DNA VAF estimations and the high VAF variability by introducing variant probability, v_PR_, a high-level descriptor of VAF distributions in chromosomal segments (continuous multi-SNV genomic regions).

v_PR_ is a parameter of a stochastic model of VAF distributions that allows for the generation of synthetic VAF samples that closely resembles the observed data. The simplicity and transparency of v_PR_ is one of the biggest advantages of the presented methodology over other existing methods.

Using variant probability, we analyzed relationships between DNA and RNA VAF estimations and biological processes. We observed that, in chromosomes affected by deletions and amplifications, VAF_RNA_ and VAF_DNA_ showed highly concordant breakpoint calls. This indicates that VAF_RNA_ alone can serve as preliminary indicator for break points of DNA deletions or amplifications if they fall within the regions covered by sequencing, and potential could facilitate the estimation of CNAs from RNA-sequencing data. Furthermore, a large proportion of v_PR_ estimates based on VAF_RNA_ samples are higher than v_PR_ estimates based on VAF_DNA_ indicating preferential transcription of some alleles in a number of chromosomal segments. Finally, we showcased that matched v_PR,NEX_ and v_PR,TEX_ values can be used to model the proportions of normal and tumor populations, thereby providing an estimate of the tumor purity. The purity estimates based on variant probabilities show good concordance with other approaches (Pearson's correlation between 0.44 and 0.76; as illustrated in Figure 9). Additionally, once the mixture composition is estimated, v_PR_ values allow for the interrogation of genetic events in each population at a specific chromosomal segment (as illustrated in Figure 5).

Since VAF estimations can be affected by allele mapping bias (Degner et al., 2009) which can lead to overestimation of the reference allele count (Brandt et al., 2015), we suggest that GetAllele input is generated from SNV-aware alignments, which perform better in VAF-based downstream analyses (Spurr et al., 2020). We note that SNV-aware alignments are now facilitated by recent methodological advances, including the implementation of the WASP method (Van De Geijn et al., 2015) in the STAR aligner (Dobin et al., 2013).

Based on our results, variant probabilities can serve as a dependable descriptor of VAF distribution and can be used to assess allele asymmetries or to aid in making matched calls of genomic events in sequencing RNA and DNA datasets without limitations caused by their different molecular nature. Finally, v_PR_ provides conceptual and mechanistic insights into relationships between VAF distributions and underlying genetic events.

Methods for estimating and analyzing v_PR_ values are implemented in a GeTallele toolbox. GeTallele allows to analyse and visualize patterns observed in the VAF distributions at a desired resolution, such as the chromosome, gene or other custom genomic level.

## Data Availability Statement

The data analyzed in this study is subject to the following licenses/restrictions: The datasets used and/or analyzed during the current study are available from the corresponding author on reasonable request. Requests to access these datasets should be directed to p.m.slowinski@exeter.ac.uk.

## Author Contributions

PS, ML, PR, NA, LS, CM, KT-A, and AH conception and design of the work. ML data acquisition. PS data analysis. PS, ML, PR, LS, KT-A, and AH interpretation of data. PS creation of new software used in the work. PS, ML, PR, LS, KT-A, and AH have drafted the work or substantively revised it. All authors approved the submitted version. All authors agreed both to be personally accountable for the author's own contributions and to ensure that questions related to the accuracy or integrity of any part of the work, even ones in which the author was not personally involved, are appropriately investigated, resolved, and the resolution documented in the literature.

## Conflict of Interest

The authors declare that the research was conducted in the absence of any commercial or financial relationships that could be construed as a potential conflict of interest.

## Abbreviations

BRCA: breast invasive carcinoma
CDF: cumulative distribution function
CNA: copy number alterations
CNA_DELETION_: copy number alterations corresponding to deletions (see section Correlation between v_PR_ and CNA)
CNA_AMPLIFICATION_: copy number alterations corresponding to amplifications (see section Correlation between v_PR_ and CNA)
CPE: consensus purity estimate
DNA: genome
EMD: earth mover's distance
FWER: family-wise error rate
FDR: false discovery rate
MEA: mean absolute error
Nex: normal exome
Ntr: normal transcriptome
r_TEX,CNA,DEL_: Pearson's correlation coefficient between v_PR,TEX_ and CNA_DELETION_
r_TEX,CNA,AMPl_: Pearson's correlation coefficient between v_PR,TEX_ and CNA_AMPLIFICATION_
r_TTR,CNA,DEL_: Pearson's correlation coefficient between v_PR,TTR_ and CNA_DELETION_
r_TTR,CNA,AMPL_: Pearson's correlation coefficient between v_PR,TTR_ and CNA_AMPLIFICATION_
p_FDR_: *p*-value after multiple comparisons Benjamini and Hochberg false discovery rate correction
PDF: probability density function
QN (e.g., Q50): N-th percentile
RNA: transcriptome
SNV: single-nucleotide variant
TCGA: the cancer genome atlas
Tex: tumor exome
Ttr: tumor transcriptome
VAF: variant allele frequency
VAF_TEX_: variant allele frequency in tumor exome sequence
VAF_TTR_: variant allele frequency in tumor transcriptome sequence
VBP: v_PR_ based purity
v_PR_: variant probability
v_PR,TEX_: variant probability estimated from tumor exome sequence
v_PR,TTR_: variant probability estimated from tumor transcriptome sequence
WES: whole exome sequencing.
