# Supplementary material for: GeTallele: A Method for Analysis of DNA and RNA Allele Frequency Distributions
Source: Front Bioeng Biotechnol. 2020 Sep 16;8:1021. doi: 10.3389/fbioe.2020.01021 (PMC7525018; doi:10.3389/fbioe.2020.01021)
Supplement: Supplementary file 1 [file Table_1.pdf]

Supplementary Table 1. Datasets, signals and purity estimates.

#	TCGA BRCA datasets	EST	ABS	LUMP	IHC	CPE
1	001_Nex_BRCA_TCGA-BH-A1FC-11A_413b80f6-f6cf-4992-804a-f045e38cbe6f<br>001_Ntr_BRCA_TCGA-BH-A1FC-11A_086db136-f3f2-42fa-aca1-63847de6ccb9<br>001_Tex_BRCA_TCGA-BH-A1FC-01A_1a2187a6-aea8-4096-8c3f-208a8467cd5a<br>001_Ttr_BRCA_TCGA-BH-A1FC-01A_b5e2f568-e6fc-4192-a3ab-da956e5bfa4c	0.7615	0.49	0.6693	0.9	0.6517
2	002_Nex_BRCA_TCGA-BH-A0B5-11A_c724807c-d80d-4582-8238-8339397b6aec<br>002_Ntr_BRCA_TCGA-BH-A0B5-11A_f478930d-216a-40ec-b434-bfc3a7b2f62b<br>002_Tex_BRCA_TCGA-BH-A0B5-01A_803de3d6-895f-4ad1-a86c-6f72d6ea8430<br>002_Ttr_BRCA_TCGA-BH-A0B5-01A_37175dfe-e34e-4f97-88b1-c0ba4bd5d093	0.7539	0.5	0.8944	0.575	0.6566
3	003_Nex_BRCA_TCGA-BH-A0BJ-11A_a9988fbb-090a-4363-bf73-7505e1710623<br>003_Ntr_BRCA_TCGA-BH-A0BJ-11A_2ced85bc-852a-4056-ad11-2e88ec6d2d82<br>003_Tex_BRCA_TCGA-BH-A0BJ-01A_58ec1111-c932-49ea-9327-1c64dfc2afa6<br>003_Ttr_BRCA_TCGA-BH-A0BJ-01A_73442f2d-3453-42ee-b57a-86871e2e2fd9	0.7386	0.37	0.8453	0.7	0.7458
4	004_Nex_BRCA_TCGA-E2-A158-11A_58fe3067-8198-486e-b0b2-286dc4451c39<br>004_Ntr_BRCA_TCGA-E2-A158-11A_323eb80d-71e2-4223-b471-a83ee42e6e08<br>004_Tex_BRCA_TCGA-E2-A158-01A_0329fa7e-d768-4bbe-940e-36f0b9829d7c<br>004_Ttr_BRCA_TCGA-E2-A158-01A_9d31f395-85e7-4ad8-95a3-0cc796c4b81d	0.9534	-	-	0.8	0.7799
5	005_Nex_BRCA_TCGA-A7-A13E-11A_bd7e6f8f-7213-4ded-a8ca-3c73c7b8d918<br>005_Ntr_BRCA_TCGA-A7-A13E-11A_99c08ce4-6526-4982-9bc7-b9c07972bcdb<br>005_Tex_BRCA_TCGA-A7-A13E-01A_28b8b84b-ca69-4c6a-860c-989777b18d32<br>005_Ttr_BRCA_TCGA-A7-A13E-01A_148d5aec-6026-46b5-b40c-38a1198175ab	0.909	0.83	0.9772	0.85	0.9184
6	006_Nex_BRCA_TCGA-BH-A208-11A_645b786f-1942-4cce-973b-4a75956265f5<br>006_Ntr_BRCA_TCGA-BH-A208-11A_a6dd96f4-f194-4c8d-8757-9e8b35465a9f<br>006_Tex_BRCA_TCGA-BH-A208-01A_5bdbd7db-ced4-4446-9069-c44c9c1f0ae0<br>006_Ttr_BRCA_TCGA-BH-A208-01A_794bcf95-8e66-4f91-a49c-ab10defe73c5	0.5951	0.31	0.6877	0.6	0.5642
7	007_Nex_BRCA_TCGA-BH-A1FU-11A_db7e821b-a2b6-40e1-9fbc-c72231b703a4<br>007_Ntr_BRCA_TCGA-BH-A1FU-11A_c051b92b-8e11-4623-b11b-3a0d52710663<br>007_Tex_BRCA_TCGA-BH-A1FU-01A_cb37bb7f-8fb6-432a-a58a-f8178d5baa64<br>007_Ttr_BRCA_TCGA-BH-A1FU-01A_7ea95c3a-b1a6-4658-b4c2-f35f3f48394e	0.6835	0.25	0.667	0.6	0.6121
8	008_Nex_BRCA_TCGA-BH-A0AY-11A_2ecc0325-3973-48b3-b53b-bb52aea5a9bc<br>008_Ntr_BRCA_TCGA-BH-A0AY-11A_1b2877ac-94a0-464c-b58b-9ce2f16aff37<br>008_Tex_BRCA_TCGA-BH-A0AY-01A_357ccb95-03e5-49f6-ab18-38d4c8d4d820<br>008_Ttr_BRCA_TCGA-BH-A0AY-01A_a19a60e7-e5ca-4f66-96fe-c9add702177d	0.6376	0.42	-	0.7	0.5612
9	009_Nex_BRCA_TCGA-BH-A18U-11A_bf3d62cb-f3a6-45d6-b9c3-416e58f1d319<br>009_Ntr_BRCA_TCGA-BH-A18U-11A_9d4c1d7e-dd77-41d1-b1df-144e7afb2141<br>009_Tex_BRCA_TCGA-BH-A18U-01A_a80933e5-3b07-41dc-b7f0-499d63c071a9<br>009_Ttr_BRCA_TCGA-BH-A18U-01A_ff89e0d9-7e6c-4b6b-a1c3-f800aaa414a1	0.7949	0.68	-	0.75	0.8077
10	010_Nex_BRCA_TCGA-AC-A2FF-11A_714e11fb-be71-4bbd-9327-457883a07ef0<br>010_Ntr_BRCA_TCGA-AC-A2FF-11A_4d32c4fa-959e-41cf-b837-104290bab9fa<br>010_Tex_BRCA_TCGA-AC-A2FF-01A_5c6fe1fc-839c-422a-89e7-4a54dcfad6c2<br>010_Ttr_BRCA_TCGA-AC-A2FF-01A_37bf962c-b180-4cc2-8e0b-fde78b4f99f4	0.5705	-	0.6868	0.8	0.6667
11	011_Nex_BRCA_TCGA-BH-A0BQ-11A_a5bdd116-8c1b-4787-be01-4c0f96709cc5<br>011_Ntr_BRCA_TCGA-BH-A0BQ-11A_45e17d22-fbed-418b-97fc-7104e1deeac1<br>011_Tex_BRCA_TCGA-BH-A0BQ-01A_27138381-1865-4a6a-bd70-58725c92cb49<br>011_Ttr_BRCA_TCGA-BH-A0BQ-01A_8879454d-b803-40b6-b3d7-fbc295de9df6	0.6814	0.4	-	0.5	0.5779
12	012_Nex_BRCA_TCGA-BH-A0BA-11A_9dbc7f19-30bd-48fd-8d5a-ca67dc26c5b1<br>012_Ntr_BRCA_TCGA-BH-A0BA-11A_2cc17895-0a6e-4703-8164-7034f5c2e1a8<br>012_Tex_BRCA_TCGA-BH-A0BA-01A_b4c0df66-54c1-4bbf-9a3c-d2fd28d5bb4b<br>012_Ttr_BRCA_TCGA-BH-A0BA-01A_a9f9701c-6b4b-48ed-af83-94804fb098a8	0.7944	0.48	0.8945	0.87	0.7278
13	013_Nex_BRCA_TCGA-BH-A0B8-11A_ef67ace2-01d6-4e8b-92c7-7c4e1e5ca327<br>013_Ntr_BRCA_TCGA-BH-A0B8-11A_3a833d6d-75c7-4381-8cef-699c633b64e6<br>013_Tex_BRCA_TCGA-BH-A0B8-01A_54972439-f9da-497d-a605-24e9670021ad<br>013_Ttr_BRCA_TCGA-BH-A0B8-01A_9c7776d0-33df-4bd7-a720-807c650fdbc5	0.8571	0.87	0.9827	0.85	0.9342
14	014_Nex_BRCA_TCGA-BH-A0AU-11A_15483d36-ad24-4771-a991-8a8435effc6a<br>014_Ntr_BRCA_TCGA-BH-A0AU-11A_7f667d91-04aa-48e8-b675-9d99b64b2058<br>014_Tex_BRCA_TCGA-BH-A0AU-01A_e7a641f3-cc31-4319-a04b-75c42e991711<br>014_Ttr_BRCA_TCGA-BH-A0AU-01A_23e09239-bfc3-4c2e-b690-db940d5292f7	0.765	0.46	0.8525	0.775	0.652
15	015_Nex_BRCA_TCGA-BH-A18S-11A_9e6d6a2d-ce9e-4d44-9603-f843ffa06c63<br>015_Ntr_BRCA_TCGA-BH-A18S-11A_b54a0f88-21be-4c6d-a27a-1c1b8959652c<br>015_Tex_BRCA_TCGA-BH-A18S-01A_d4746397-9268-460a-954b-e5b5921138f9<br>015_Ttr_BRCA_TCGA-BH-A18S-01A_e0a3ea3a-ffce-4e30-9f42-cb047a7644a1	0.8948	0.89	-	0.85	0.8676

16	016_Nex_BRCA_TCGA-BH-A0HK-11A_d256dce0-d74b-4f8f-bf47-40b1b953fc7f<br>016_Ntr_BRCA_TCGA-BH-A0HK-11A_438650e8-0ee2-4c74-8432-88b5c8006187<br>016_Tex_BRCA_TCGA-BH-A0HK-01A_944b4c29-bf72-4eec-b277-badc237730de<br>016_Ttr_BRCA_TCGA-BH-A0HK-01A_fe04f368-0a73-4f97-9d6b-2986f9b2b052	0.7649	0.78	0.9163	0.925	0.8357
17	017_Nex_BRCA_TCGA-A7-A0D9-11A_dda70534-0d4d-4c30-9c6a-fb3c39396fb0<br>017_Ntr_BRCA_TCGA-A7-A0D9-11A_17cf6364-e228-4ee9-bffa-d1ad75f4152b<br>017_Tex_BRCA_TCGA-A7-A0D9-01A_821d7a33-77fb-496e-be9c-0552b12cbbee<br>017_Ttr_BRCA_TCGA-A7-A0D9-01A_c0ecd314-9d99-48ec-83f1-5a0c1ed656aa	0.8911	0.8	1	0.775	0.8921
18	018_Nex_BRCA_TCGA-BH-A0BV-11A_56dfc492-2b1f-4494-9ba9-14a70601ae21<br>018_Ntr_BRCA_TCGA-BH-A0BV-11A_20459115-d7be-4d04-896f-c5ff6923ec4c<br>018_Tex_BRCA_TCGA-BH-A0BV-01A_beb9e4cf-1f76-4a26-acee-e88d0936e60b<br>018_Ttr_BRCA_TCGA-BH-A0BV-01A_d037d3c2-e316-473d-9970-d4fb43615d95	0.6895	0.54	-	0.725	0.6749
19	019_Nex_BRCA_TCGA-E2-A1LH-11A_61558dd3-8f6c-4f70-8717-7676580fa5a7<br>019_Ntr_BRCA_TCGA-E2-A1LH-11A_c7e02b93-465f-47da-81d7-ec9a8cb1e52b<br>019_Tex_BRCA_TCGA-E2-A1LH-01A_f54770bb-5dd0-48cf-ac5a-3f023a6aef95<br>019_Ttr_BRCA_TCGA-E2-A1LH-01A_169c390c-a211-4db0-a983-9bf5d6eee16e	0.5948	0.32	0.5637	0.8	0.4633
20	020_Nex_BRCA_TCGA-BH-A0DD-11A_e9fe9b97-f7c7-40dc-ae31-17bb15c9fd8b<br>020_Ntr_BRCA_TCGA-BH-A0DD-11A_5482cdd0-3698-455b-97c1-b10c69d67ae9<br>020_Tex_BRCA_TCGA-BH-A0DD-01A_99ca9706-f2bf-430b-9b23-e0947c0f8593<br>020_Ttr_BRCA_TCGA-BH-A0DD-01A_90cbc532-1ca8-46d6-977c-72b6d01e9c34	0.8677	0.79	0.9459	0.625	0.8714
21	021_Nex_BRCA_TCGA-BH-A0H5-11A_adfb1a86-fbb1-4b71-9c04-f99399f20d70<br>021_Ntr_BRCA_TCGA-BH-A0H5-11A_896d76a1-bae8-495a-9e12-e82e16bd8b16<br>021_Tex_BRCA_TCGA-BH-A0H5-01A_6cc3c90e-c77c-4609-ada5-9b78c659dc34<br>021_Ttr_BRCA_TCGA-BH-A0H5-01A_778c9326-998d-4081-b148-0eede2b94e29	0.4399	-	-	0.475	0.1632
22	022_Nex_BRCA_TCGA-A7-A0DB-11A_91081819-79c8-4de6-bfdb-742df760c08b<br>022_Ntr_BRCA_TCGA-A7-A0DB-11A_a8ed2ec3-0285-4028-9698-710a148ce11b<br>022_Tex_BRCA_TCGA-A7-A0DB-01A_37a9daca-9d53-4ec4-8de2-dc2c140a5d8f<br>022_Ttr_BRCA_TCGA-A7-A0DB-01A_1f62e969-d05d-4a4d-a163-cb06e4958f71	0.7341	0.44	-	0.85	0.6494
23	023_Nex_BRCA_TCGA-BH-A1FN-11A_e1c0d95f-949c-4cec-9cf8-f91c3b90b8d9<br>023_Ntr_BRCA_TCGA-BH-A1FN-11A_d5e5f3c9-4129-4c92-87f3-6f86577a7584<br>023_Tex_BRCA_TCGA-BH-A1FN-01A_8d715491-6943-4d58-92f6-88cce7b463e2<br>023_Ttr_BRCA_TCGA-BH-A1FN-01A_8e7dc738-8a8f-45b2-bd82-4125a07d7373	0.8367	0.7	0.8509	0.75	0.8313
24	024_Nex_BRCA_TCGA-BH-A0AZ-11A_9bbae9a0-9f12-48cf-9aa7-d070c6627ea5<br>024_Ntr_BRCA_TCGA-BH-A0AZ-11A_693bf8e4-b266-4b58-b812-f579179efb65<br>024_Tex_BRCA_TCGA-BH-A0AZ-01A_664528b7-b511-4627-8464-0702263434c5<br>024_Ttr_BRCA_TCGA-BH-A0AZ-01A_07f377f4-0bd1-4647-bf06-ff6ed553c44a	0.6505	0.53	0.8696	0.7	0.6655
25	025_Nex_BRCA_TCGA-BH-A0HA-11A_c61bb1ab-688f-4d58-8388-60ae77c28840<br>025_Ntr_BRCA_TCGA-BH-A0HA-11A_09e07a68-a443-4c16-a0de-78cd8aea59c0<br>025_Tex_BRCA_TCGA-BH-A0HA-01A_2c144eba-6490-4d64-9446-085d6edc8308<br>025_Ttr_BRCA_TCGA-BH-A0HA-01A_6d483def-2d91-4afc-991a-4a29804a6f3a	0.6418	0.74	0.9258	0.725	0.7386
26	026_Nex_BRCA_TCGA-A7-A0CE-11A_eee8d4d0-d524-47f5-b076-6ad6216de1a3<br>026_Ntr_BRCA_TCGA-A7-A0CE-11A_548cad87-ec95-47e2-890e-7c8284ea5b88<br>026_Tex_BRCA_TCGA-A7-A0CE-01A_4288da4e-7e77-434b-a092-9450b0cb7833<br>026_Ttr_BRCA_TCGA-A7-A0CE-01A_14201682-0c8d-49c7-a5e1-7026e1a07b69	0.9035	0.73	-	0.835	0.8551
27	027_Nex_BRCA_TCGA-BH-A0DK-11A_3f4400a1-84ab-4198-b9a1-67b2ffc5ef36<br>027_Ntr_BRCA_TCGA-BH-A0DK-11A_ae67044f-62c9-405f-bfc1-f0b8f1bc66d3<br>027_Tex_BRCA_TCGA-BH-A0DK-01A_e3e2053a-3ca2-4527-9b94-209def68dcc3<br>027_Ttr_BRCA_TCGA-BH-A0DK-01A_a3df35ec-a8d2-44ad-8ba6-eaba504261e0	0.5384	0.53	0.7316	0.7	0.6837
28	028_Nex_BRCA_TCGA-BH-A0E1-11A_f6fed4ed-a853-40aa-bf7b-e627efd402d6<br>028_Ntr_BRCA_TCGA-BH-A0E1-11A_52441de4-e26b-42b3-b061-94907c049501<br>028_Tex_BRCA_TCGA-BH-A0E1-01A_3c7e6a59-08b8-4903-932a-99946a96b746<br>028_Ttr_BRCA_TCGA-BH-A0E1-01A_f412f8d8-9e35-41d9-b44a-131186cb4bb0	0.8676	0.75	0.9742	0.825	0.8524
29	029_Nex_BRCA_TCGA-BH-A0DG-11A_c99b1fb3-17e3-4472-86ee-7fda358a92c2<br>029_Ntr_BRCA_TCGA-BH-A0DG-11A_bfdaf242-1e97-450d-9983-2cbb4e99305d<br>029_Tex_BRCA_TCGA-BH-A0DG-01A_721e2f71-60ae-4d63-9f05-113bce56c672<br>029_Ttr_BRCA_TCGA-BH-A0DG-01A_865afd6b-84a7-4dde-aa23-0b925c0b9d50	0.6358	0.42	0.7804	0.775	0.5386
30	030_Nex_BRCA_TCGA-AC-A2FB-11A_552279ea-d7b1-496d-8170-ca30f5b62b5a<br>030_Ntr_BRCA_TCGA-AC-A2FB-11A_56cd7da0-2c47-4986-91ce-07db2bb87369<br>030_Tex_BRCA_TCGA-AC-A2FB-01A_de000c35-8bf4-470a-9656-1b5da0deebe6<br>030_Ttr_BRCA_TCGA-AC-A2FB-01A_35aa5078-e07f-4a0f-84c1-01a0e566e97c	0.5372	0.23	0.5493	0.7	0.4436

31	031_Nex_BRCA_TCGA-BH-A0H7-11A_abfca562-d328-40d2-83bb-e584123b0f28<br>031_Ntr_BRCA_TCGA-BH-A0H7-11A_d969d9b2-9d8b-4594-95d4-87e6ce1236fc<br>031_Tex_BRCA_TCGA-BH-A0H7-01A_e8daad78-39fc-4835-b1c4-8807653d9c9a<br>031_Ttr_BRCA_TCGA-BH-A0H7-01A_0d37f87a-760a-472a-acba-bbc255422fbe	0.7939	0.63	0.9561	0.725	0.7534
32	032_Nex_BRCA_TCGA-BH-A1EU-11A_38e87966-9605-4454-a4d1-28f96b7689f7<br>032_Ntr_BRCA_TCGA-BH-A1EU-11A_3b00c121-17f2-461e-8873-08d15c9ec9f4<br>032_Tex_BRCA_TCGA-BH-A1EU-01A_4bccbb0f-2641-44df-b89a-42f020b4c08f<br>032_Ttr_BRCA_TCGA-BH-A1EU-01A_86e3dba1-48fb-44cc-b046-8bc35963ce99	0.5387	0.33	0.6869	0.65	0.4299
33	033_Nex_BRCA_TCGA-BH-A0DP-11A_27543260-52ac-444b-8214-e62dca2cc8fe<br>033_Ntr_BRCA_TCGA-BH-A0DP-11A_30f4e5d8-a13d-4ef2-88e0-a01e07c2e142<br>033_Tex_BRCA_TCGA-BH-A0DP-01A_0326975a-2e56-404a-8776-92c5c5678853<br>033_Ttr_BRCA_TCGA-BH-A0DP-01A_7ff8a7a0-5235-4de0-bb9f-b811230b5bda	0.7037	0.42	0.8565	0.7	0.655
34	034_Nex_BRCA_TCGA-BH-A18N-11A_6c8aae77-5f43-41ec-a139-81f3ba02f6ea<br>034_Ntr_BRCA_TCGA-BH-A18N-11A_0738f1b2-aa50-4921-82e1-d3614b40f98d<br>034_Tex_BRCA_TCGA-BH-A18N-01A_b6f89799-9070-4fbc-b10c-53cbe515eccc<br>034_Ttr_BRCA_TCGA-BH-A18N-01A_4b7b8eb8-d939-411c-be5e-cf41a5521963	0.8685	0.76	-	0.9	0.832
35	035_Nex_BRCA_TCGA-BH-A0BC-11A_6359db46-f8dd-4dc8-a3a9-8725d8f6958a<br>035_Ntr_BRCA_TCGA-BH-A0BC-11A_2cb50d4a-d6df-4b64-acfb-7a7db5ddd1de<br>035_Tex_BRCA_TCGA-BH-A0BC-01A_73b9208d-336c-4990-a27d-0164a77dd165<br>035_Ttr_BRCA_TCGA-BH-A0BC-01A_92e26b53-f540-428a-a3c5-848a36b31171	0.6221	0.6	0.8221	0.8	0.7727
36	036_Nex_BRCA_TCGA-BH-A0BZ-11A_2b4e3d99-07cd-4b06-ad97-82a19ac0eb5d<br>036_Ntr_BRCA_TCGA-BH-A0BZ-11A_3aa16a4b-4e35-4530-84fb-0cb204290b08<br>036_Tex_BRCA_TCGA-BH-A0BZ-01A_74414845-839f-4885-b13d-3f2e17781f84<br>036_Ttr_BRCA_TCGA-BH-A0BZ-01A_efefcc2f-72e9-4634-b943-d26083e1a312	0.5788	0.37	0.7363	0.6	0.5138
37	037_Nex_BRCA_TCGA-BH-A0DL-11A_2d495f9c-4ffa-4169-b583-6786612e9606<br>037_Ntr_BRCA_TCGA-BH-A0DL-11A_bd8b100a-8391-4046-847f-c3fdd3830eeb<br>037_Tex_BRCA_TCGA-BH-A0DL-01A_dfd355e4-478a-47cb-9aab-8ce22b6f936c<br>037_Ttr_BRCA_TCGA-BH-A0DL-01A_11d77ef2-b3f9-4af9-8490-71f9a8c599e0	0.6944	0.53	-	0.65	0.6655
38	038_Nex_BRCA_TCGA-BH-A0BT-11A_32430467-5215-4738-86a3-5bbe11fbba86<br>038_Ntr_BRCA_TCGA-BH-A0BT-11A_cbef4196-5f3b-40d9-b26f-5b2bb82fbe9b<br>038_Tex_BRCA_TCGA-BH-A0BT-01A_e78b9962-7bc2-4238-806a-5933ac07de99<br>038_Ttr_BRCA_TCGA-BH-A0BT-01A_aae75165-efa0-46b3-8a8d-82dc7d82aecd	0.8096	0.67	0.9054	0.75	0.7751
39	039_Nex_BRCA_TCGA-BH-A18Q-11A_b58d4f69-a4ea-489b-9d25-e5cfdc465adb<br>039_Ntr_BRCA_TCGA-BH-A18Q-11A_76575097-374b-4fb2-8054-2a31b4204165<br>039_Tex_BRCA_TCGA-BH-A18Q-01A_1f2c90ef-a05d-494c-9232-e705691f46b9<br>039_Ttr_BRCA_TCGA-BH-A18Q-01A_f0173e28-7fe4-411f-a187-57fd94a7935a	0.8117	0.73	-	0.9	0.836
40	040_Nex_BRCA_TCGA-E2-A1LB-11A_e2d7a695-b0bf-4432-8f98-1843bb49efba<br>040_Ntr_BRCA_TCGA-E2-A1LB-11A_6eb518ab-f174-45ae-8d65-74086ecb1125<br>040_Tex_BRCA_TCGA-E2-A1LB-01A_3ddbc444-ee1b-43be-bab5-b0f67d5eb339<br>040_Ttr_BRCA_TCGA-E2-A1LB-01A_d7d566a0-b6d0-4a4f-8211-9309b27b0ade	0.8415	0.68	0.8192	0.9	0.815
41	041_Nex_BRCA_TCGA-BH-A0DH-11A_a7a7e0f6-100f-4145-9599-693e6c14e903<br>041_Ntr_BRCA_TCGA-BH-A0DH-11A_5a0374e5-cee9-4952-9df0-4ff125196478<br>041_Tex_BRCA_TCGA-BH-A0DH-01A_eb680f8c-4ba1-45ef-8b94-e58b68922f2f<br>041_Ttr_BRCA_TCGA-BH-A0DH-01A_71a3c27c-0982-4da6-b260-cf16a4868a19	0.8317	0.76	0.8955	0.85	0.8597
42	042_Nex_BRCA_TCGA-BH-A0B7-11A_d9aca915-ea30-4939-af59-edaef8872396<br>042_Ntr_BRCA_TCGA-BH-A0B7-11A_8db8b247-05b8-46ca-8791-ecf846da2c7f<br>042_Tex_BRCA_TCGA-BH-A0B7-01A_e3b9eb8a-93f3-4668-a54b-fa8b15be5667<br>042_Ttr_BRCA_TCGA-BH-A0B7-01A_0fdae4ee-ca68-4ba4-ba58-76058409b02f	0.6207	0.2	-	0.575	0.4954
43	043_Nex_BRCA_TCGA-E2-A15I-11A_36024763-f828-4496-8fdc-46d5c3de569b<br>043_Ntr_BRCA_TCGA-E2-A15I-11A_ffa9ace8-9253-4775-9ad2-2a8a50c0f9c9<br>043_Tex_BRCA_TCGA-E2-A15I-01A_8c627466-eb99-4a7e-87e6-314ae8ed32a1<br>043_Ttr_BRCA_TCGA-E2-A15I-01A_3a4e3785-fb2e-4ffc-9644-91c78a9a9ebe	0.7689	0.62	0.768	0.8	0.7565
44	044_Nex_BRCA_TCGA-BH-A0DV-11A_79a92eab-c87c-4209-819e-193d653c0df6<br>044_Ntr_BRCA_TCGA-BH-A0DV-11A_e87e7e3e-9059-47cf-9f45-8959250b037f<br>044_Tex_BRCA_TCGA-BH-A0DV-01A_105290eb-b626-4318-9b8a-42f477e2cec6<br>044_Ttr_BRCA_TCGA-BH-A0DV-01A_7bad3f4c-6065-4245-8119-c25596f38829	0.6021	0.31	0.7631	0.7	0.4856
45	045_Nex_BRCA_TCGA-BH-A0DZ-11A_aebf04d4-4a1b-4a50-b5f1-0f9e2c273121<br>045_Ntr_BRCA_TCGA-BH-A0DZ-11A_80b4d43d-9e7d-4ab8-b05a-0eb51faa9d12<br>045_Tex_BRCA_TCGA-BH-A0DZ-01A_4e7d62f5-4be9-4b9c-9b7c-aec4567dded2<br>045_Ttr_BRCA_TCGA-BH-A0DZ-01A_8b1982a0-315c-47e1-8de0-a1e5ec51dd74	0.6572	0.65	-	0.885	0.7792
46	046_Nex_BRCA_TCGA-BH-A18R-11A_b32b2067-a79e-42c5-ae78-135c845253fe<br>046_Ntr_BRCA_TCGA-BH-A18R-11A_f82099ae-9d74-44d8-ba5b-cd10eeb09807	0.8685	0.53	-	0.85	0.7466

	046_Tex_BRCA_TCGA-BH-A18R-01A_c518bc34-50dc-4265-824f-a954e4d19f0b<br>046_Ttr_BRCA_TCGA-BH-A18R-01A_fc65ff2e-9808-4c1e-a16b-8285fd0d27df					
47	047_Nex_BRCA_TCGA-E2-A15K-11A_6299f114-932a-42c0-8cab-bebb12c996fc<br>047_Ntr_BRCA_TCGA-E2-A15K-11A_c2ab9488-d9a4-479c-b9e2-6f9b0cdbaacb<br>047_Tex_BRCA_TCGA-E2-A15K-01A_80019ec7-b0d8-4573-b5d6-a5d9f2745ab2<br>047_Ttr_BRCA_TCGA-E2-A15K-01A_7e3a600b-edd8-428e-b88d-af4c63dcaad9	0.7425	0.7	0.724	0.9	-
48	048_Nex_BRCA_TCGA-BH-A1EN-11A_a6259119-3d8a-4749-a517-c675efbc8215<br>048_Ntr_BRCA_TCGA-BH-A1EN-11A_488f1b69-c2a3-429b-a972-31edfd615a67<br>048_Tex_BRCA_TCGA-BH-A1EN-01A_72e4cb26-911c-4804-9e4f-ed5b51024cd1<br>048_Ttr_BRCA_TCGA-BH-A1EN-01A_96360e75-26b6-4647-b974-9e31ae6de00c	0.8518	0.7	0.9612	0.85	0.8179
49	049_Nex_BRCA_TCGA-BH-A0H9-11A_d8b452e5-010a-4fec-80a4-770a5a492090<br>049_Ntr_BRCA_TCGA-BH-A0H9-11A_1337ceba-db77-4b31-ac20-1c6a8bb5f546<br>049_Tex_BRCA_TCGA-BH-A0H9-01A_ac4899fe-f56d-4b98-9a54-73ffd0c90652<br>049_Ttr_BRCA_TCGA-BH-A0H9-01A_a97d281f-235f-481b-b26b-169b96e0e65f	0.8872	0.35	0.7934	0.7	0.747
50	050_Nex_BRCA_TCGA-E2-A153-11A_1c3f2e11-952a-4e47-a8b9-25f4fe4bf205<br>050_Ntr_BRCA_TCGA-E2-A153-11A_bd0ab51c-c114-40e3-a6a1-4b8f576a41d3<br>050_Tex_BRCA_TCGA-E2-A153-01A_85258fb2-26ab-4a66-b8a5-5a58bf9275e0<br>050_Ttr_BRCA_TCGA-E2-A153-01A_091a54cd-e3b3-4af2-828f-a80e64504f5e	0.6589	0.61	-	0.6	0.5474
51	051_Nex_BRCA_TCGA-BH-A0BW-11A_ab130f7f-4070-436e-ac7b-c1b7aecb9dc6<br>051_Ntr_BRCA_TCGA-BH-A0BW-11A_2581c95b-1b57-4407-bbc4-a65c89bfd136<br>051_Tex_BRCA_TCGA-BH-A0BW-01A_7661179f-df6c-4a57-adca-224b62d98348<br>051_Ttr_BRCA_TCGA-BH-A0BW-01A_a15d171e-ac82-4712-94a9-b4799e7b2915	0.6784	0.54	-	0.6	0.6749
52	052_Nex_BRCA_TCGA-BH-A18J-11A_3aa5b173-17b2-425f-b5c7-395614d6bfa2<br>052_Ntr_BRCA_TCGA-BH-A18J-11A_307eb339-a781-45cc-9597-da0be7e5438a<br>052_Tex_BRCA_TCGA-BH-A18J-01A_f639a485-8ebb-4dcc-9f2e-a8d7ad05564f<br>052_Ttr_BRCA_TCGA-BH-A18J-01A_0e985713-0492-4191-918b-fef6c23389b1	0.8066	0.51	-	0.7	0.7243
53	053_Nex_BRCA_TCGA-BH-A204-11A_98e5c1d8-5c14-4416-8437-31d0098dd341<br>053_Ntr_BRCA_TCGA-BH-A204-11A_2afdc0cf-2723-42dd-89f7-fa03c6ba218c<br>053_Tex_BRCA_TCGA-BH-A204-01A_970600ce-9486-4641-8555-533132f7a414<br>053_Ttr_BRCA_TCGA-BH-A204-01A_893fcf87-8baa-424e-8866-bcf8cfa26cf9	0.8763	0.89	0.9364	0.85	0.9601
54	054_Nex_BRCA_TCGA-BH-A18K-11A_502ee86f-829e-4b6e-a8f1-be082c445310<br>054_Ntr_BRCA_TCGA-BH-A18K-11A_375bcdd8-8628-4047-948a-fa98bfa3dba5<br>054_Tex_BRCA_TCGA-BH-A18K-01A_7e8c2ea7-04ce-47c5-b848-229f96563015<br>054_Ttr_BRCA_TCGA-BH-A18K-01A_12473f59-359c-4306-ade3-2156e458cd05	0.8058	0.58	-	0.8	0.7468
55	055_Nex_BRCA_TCGA-BH-A0C3-11A_9fedd2f4-d2c8-4d24-987b-69edf55e15f1<br>055_Ntr_BRCA_TCGA-BH-A0C3-11A_a49fa48d-efc4-4b99-a42e-7019236af6c8<br>055_Tex_BRCA_TCGA-BH-A0C3-01A_866d9cb0-a299-46c1-a787-2e73fd758fbe<br>055_Ttr_BRCA_TCGA-BH-A0C3-01A_164f86df-dec9-44ef-b1c7-2ee5d33617be	0.6289	0.39	0.7847	0.45	0.528
56	056_Nex_BRCA_TCGA-BH-A0C0-11A_9778035c-19ff-4a89-bba2-fa83e51d9add<br>056_Ntr_BRCA_TCGA-BH-A0C0-11A_52a72824-0b41-4b8e-86f0-41ee5e00c989<br>056_Tex_BRCA_TCGA-BH-A0C0-01A_568a2363-b7e5-48f6-9242-328950eebf39<br>056_Ttr_BRCA_TCGA-BH-A0C0-01A_f5fe9655-f5b4-413b-882c-43b872e4ec23	0.6223	0.27	0.5586	0.85	0.5068
57	057_Nex_BRCA_TCGA-BH-A0E0-11A_59fa57c8-7435-4499-bd09-bf969596c18d<br>057_Ntr_BRCA_TCGA-BH-A0E0-11A_a3e5f7bd-3ab0-4ea6-9de1-742de1a2ed78<br>057_Tex_BRCA_TCGA-BH-A0E0-01A_72436fcd-21fd-46bd-bd36-7f514edb51de<br>057_Ttr_BRCA_TCGA-BH-A0E0-01A_b98d2a16-974c-4728-9648-81dc4314f225	0.9013	0.57	0.7391	0.875	0.7486
58	058_Nex_BRCA_TCGA-BH-A18M-11A_8e0cb775-9fc9-4001-973c-ef6cec2a38b6<br>058_Ntr_BRCA_TCGA-BH-A18M-11A_3e02aa37-30ee-4663-bba1-280e5127f302<br>058_Tex_BRCA_TCGA-BH-A18M-01A_69ccc418-264c-4e8e-a034-39607c07fa59<br>058_Ttr_BRCA_TCGA-BH-A18M-01A_aea8fff8-dbc9-4a1b-9a3a-ca1882432c57	0.7363	0.45	-	0.85	0.6525
59	059_Nex_BRCA_TCGA-E2-A1BC-11A_45b8b995-c477-4358-8050-6d41c267b467<br>059_Ntr_BRCA_TCGA-E2-A1BC-11A_205007dd-4bc1-4e1f-9fdb-115b0e7c9836<br>059_Tex_BRCA_TCGA-E2-A1BC-01A_f969cce4-0fcd-47bb-91f1-37ba0f314994<br>059_Ttr_BRCA_TCGA-E2-A1BC-01A_ea3bd91d-520b-4198-b011-a0f578eadc3e	0.8083	0.56	0.8676	0.85	0.7707
60	060_Nex_BRCA_TCGA-BH-A203-11A_f08939ed-a218-4688-b419-a91333d0267b<br>060_Ntr_BRCA_TCGA-BH-A203-11A_8c2d82aa-0b36-488f-8cf1-f82795b831c5<br>060_Tex_BRCA_TCGA-BH-A203-01A_55a9b84d-ca9f-402c-8d93-15aef2fde988<br>060_Ttr_BRCA_TCGA-BH-A203-01A_8986bdd8-2be5-41ed-9596-59ca1f95e1c0	0.7637	0.37	0.6872	0.75	0.5905
61	061_Nex_BRCA_TCGA-BH-A1F2-11A_d6eb2d94-1234-46b3-9403-958f3b340fd0<br>061_Ntr_BRCA_TCGA-BH-A1F2-11A_210014fa-f161-4799-a1c0-6f93d4b631f6<br>061_Tex_BRCA_TCGA-BH-A1F2-01A_91aeda5a-ed5a-4175-b19e-408219b980fc<br>061_Ttr_BRCA_TCGA-BH-A1F2-01A_ceb5a503-e107-4721-9283-714406cdd914	0.7507	0.52	0.8661	0.6	0.754

62	062_Nex_BRCA_TCGA-BH-A1EO-11A_90308930-e3c5-47bb-bcee-58eaae7d3dfa<br>062_Ntr_BRCA_TCGA-BH-A1EO-11A_a426d9c2-86b1-4db1-b49c-eceaa01273a9<br>062_Tex_BRCA_TCGA-BH-A1EO-01A_7787e3e2-f604-4b4d-a3bc-60c795d4177b<br>062_Ttr_BRCA_TCGA-BH-A1EO-01A_e31bd4a4-ecda-49b4-83b0-7f1496c2f9ae	0.5849	0.48	0.8854	0.9	0.7493
63	063_Nex_BRCA_TCGA-BH-A18V-11A_353e7fa1-08c5-400a-b352-b5325e40d66c<br>063_Ntr_BRCA_TCGA-BH-A18V-11A_e3c5cba8-e3ba-4e0b-929b-280708e0a855<br>063_Tex_BRCA_TCGA-BH-A18V-01A_abcf2a8e-6f4c-4668-9ef9-41d95d16e8e6<br>063_Ttr_BRCA_TCGA-BH-A18V-01A_286394db-7d5e-4de2-b386-581352164350	0.6941	0.56	-	0.75	-
64	064_Nex_BRCA_TCGA-BH-A0DT-11A_6dde640a-1d79-4e7d-9491-c500b8183d9a<br>064_Ntr_BRCA_TCGA-BH-A0DT-11A_71aa4cd6-75ea-4e10-b16c-ea9adbf31a98<br>064_Tex_BRCA_TCGA-BH-A0DT-01A_9d93c6fb-336a-4cb4-9f33-8557456753b1<br>064_Ttr_BRCA_TCGA-BH-A0DT-01A_61ad7408-dacd-4913-a479-c456e8b03191	0.7462	0.42	-	0.55	0.6659
65	065_Nex_BRCA_TCGA-GI-A2C9-11A_454dbdba-da53-4e99-9670-dff1e5bbb77c<br>065_Ntr_BRCA_TCGA-GI-A2C9-11A_d8aa0349-d74e-4891-8398-6476eb1935f0<br>065_Tex_BRCA_TCGA-GI-A2C9-01A_2f2b0909-488b-4fa3-8251-2ef6e7d5869e<br>065_Ttr_BRCA_TCGA-GI-A2C9-01A_01ea694e-989b-4a35-9397-5e508656d1d8	0.8062	0.51	0.8386	0.8	0.6969
66	066_Nex_BRCA_TCGA-BH-A209-11A_c580c610-832d-45be-9963-06bb918ede73<br>066_Ntr_BRCA_TCGA-BH-A209-11A_b8b48554-ca2f-466d-85b0-9d473cca8ca7<br>066_Tex_BRCA_TCGA-BH-A209-01A_7b85ca36-2fe9-4156-99eb-f79463dbc572<br>066_Ttr_BRCA_TCGA-BH-A209-01A_b2cf947a-5ed1-4e24-8752-bf2a6eca895a	0.5979	0.24	0.6243	0.6	0.4645
67	067_Nex_BRCA_TCGA-BH-A1EV-11A_f4d30842-7873-46d3-8f25-ae7d05909175<br>067_Ntr_BRCA_TCGA-BH-A1EV-11A_73e296db-9eec-4060-97c9-80a98dbb9fb6<br>067_Tex_BRCA_TCGA-BH-A1EV-01A_fb502696-cb13-487a-a70a-6ceefcf20ca0<br>067_Ttr_BRCA_TCGA-BH-A1EV-01A_93ab3adf-7ab9-455e-9007-9f51443352fe	0.8435	0.61	0.9517	0.8	0.817
68	068_Nex_BRCA_TCGA-GI-A2C8-11A_836e4482-11c7-4422-a2e5-cac9b846ea71<br>068_Ntr_BRCA_TCGA-GI-A2C8-11A_580d9a3d-e198-4e7b-aa1f-419d868bb0b5<br>068_Tex_BRCA_TCGA-GI-A2C8-01A_146c0ba4-6761-446c-be7c-7e56c0ffa37b<br>068_Ttr_BRCA_TCGA-GI-A2C8-01A_c0ee6e25-02b9-4b2f-9f23-fd61eedf9945	0.601	0.48	0.7846	0.85	0.6998
69	069_Nex_BRCA_TCGA-BH-A0BM-11A_92faafbd-6a76-4116-80fc-a15767aa81d0<br>069_Ntr_BRCA_TCGA-BH-A0BM-11A_ae127be2-5e4c-4b7e-9cf8-3aa9e529baaa<br>069_Tex_BRCA_TCGA-BH-A0BM-01A_c513ed81-255f-43b0-b8aa-984326201745<br>069_Ttr_BRCA_TCGA-BH-A0BM-01A_006b2b95-7069-4cb6-bfe8-7edb80056add	0.796	0.61	0.9189	0.84	0.7886
70	070_Nex_BRCA_TCGA-BH-A18L-11A_7a010ccd-f780-45f0-98da-cc738e87b6d3<br>070_Ntr_BRCA_TCGA-BH-A18L-11A_ef4660c0-c177-4d46-90f9-56e3dc47b59e<br>070_Tex_BRCA_TCGA-BH-A18L-01A_0d4aca9c-c11e-4f78-a250-08d45ce4828e<br>070_Ttr_BRCA_TCGA-BH-A18L-01A_1af43803-7afa-4d2b-aa78-2dec84c1e702	0.927	0.81	-	0.8	0.9113
71	071_Nex_BRCA_TCGA-A7-A13F-11A_471d1e10-7c79-44f9-a373-bd3e510b6155<br>071_Ntr_BRCA_TCGA-A7-A13F-11A_4e4cd9e5-27bb-4ea7-9328-0b267373ec1c<br>071_Tex_BRCA_TCGA-A7-A13F-01A_d8fad6b2-66b8-4d6f-b018-653998675921<br>071_Ttr_BRCA_TCGA-A7-A13F-01A_75898a6d-75e4-4dca-a7ed-c11056e0c9c4	0.8478	0.65	0.9236	0.7	0.7915
72	072_Nex_BRCA_TCGA-E2-A15M-11A_b2138cda-519f-4691-bf1c-0f863b55d888<br>072_Ntr_BRCA_TCGA-E2-A15M-11A_bf873756-8ee8-49bd-b2ca-17223c7ef962<br>072_Tex_BRCA_TCGA-E2-A15M-01A_1ccf392d-7959-4bb9-8ca8-4298409f4951<br>072_Ttr_BRCA_TCGA-E2-A15M-01A_b2569032-6147-4a1c-973f-d5985127e9f4	0.4911	0.28	-	0.8	0.4285
