# Supplementary material for: GeTallele: A Method for Analysis of DNA and RNA Allele Frequency Distributions
Source: Front Bioeng Biotechnol. 2020 Sep 16;8:1021. doi: 10.3389/fbioe.2020.01021 (PMC7525018; doi:10.3389/fbioe.2020.01021)
Supplement: Supplementary file 2 [file Image_1.pdf]

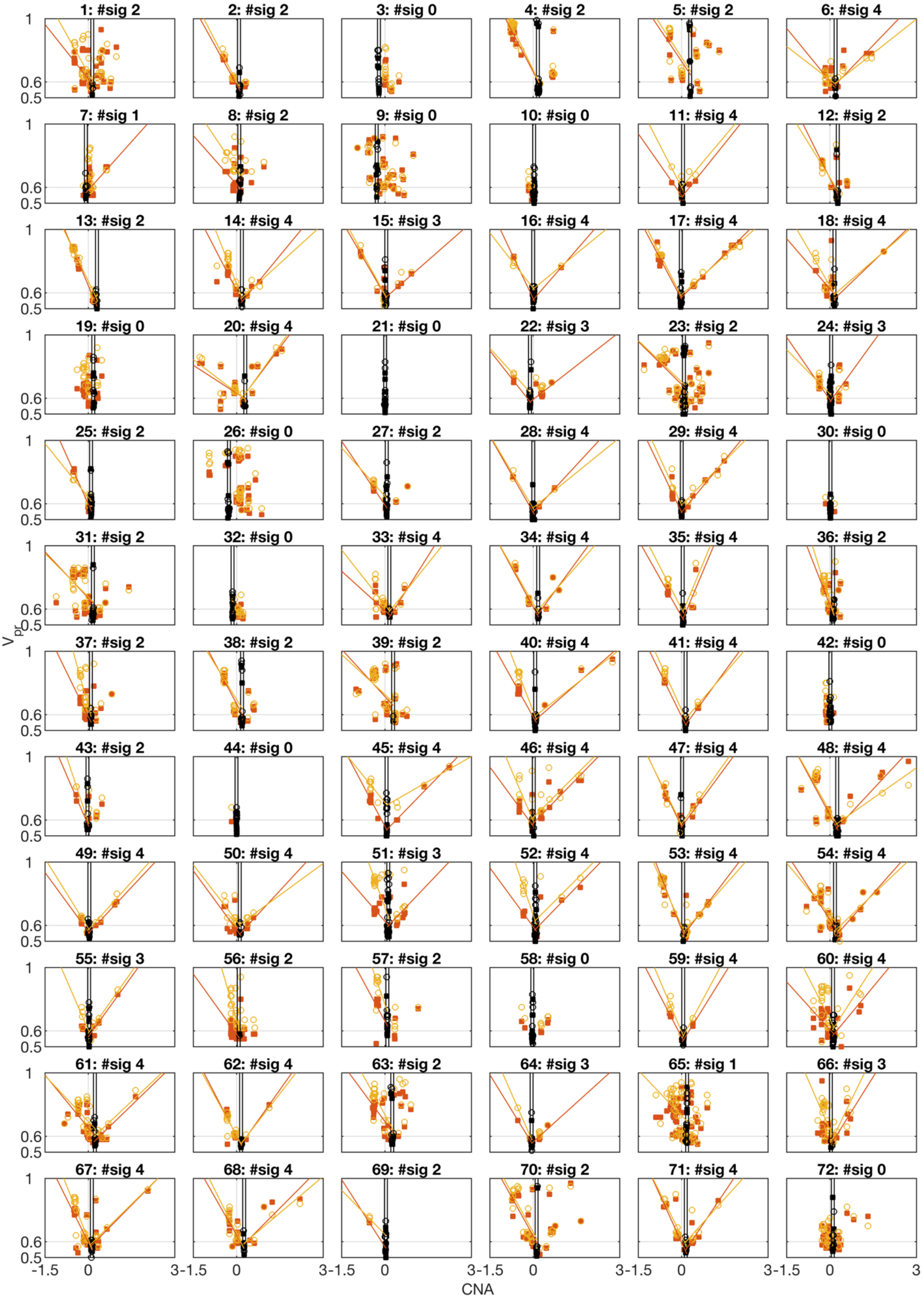

Supplementary Fig. 1. Illustration of the correlations between v_{PR} and CNA. Orange squares $v_{PR,TEX}$, yellow circles $v_{PR,TTR}$. Lines, least-squares fitted trends for significant correlations (orange correlation with $v_{PR,TEX}$, yellow correlation with $v_{PR,TTR}$). Black, v_{PR} for $CNA_{MIN}\pm0.05$. Title format Number of the dataset: #sig number of significant correlations in the dataset.
